# Probe-dependent Proximity Profiling (ProPPr) Uncovers Similarities and Differences in Phospho-Tau-Associated Proteomes Between Tauopathies

**DOI:** 10.1186/s13024-025-00817-0

**Published:** 2025-03-13

**Authors:** Dmytro Morderer, Melissa C. Wren, Feilin Liu, Naomi Kouri, Anastasiia Maistrenko, Bilal Khalil, Nora Pobitzer, Michelle R. Salemi, Brett S. Phinney, Guojun Bu, Na Zhao, Dennis W. Dickson, Melissa E. Murray, Wilfried Rossoll

**Affiliations:** 1 Department of Neuroscience, Mayo Clinic, Jacksonville, FL USA; 2 Proteomics Core, University of California Davis, Davis, CA USA; 3 Present address: Division of Life Science, The Hong Kong University of Science and Technology, Clear Water Bay, Hong Kong, China

**Keywords:** Tauopathy, Proximity labeling, Proteomics, Mass spectrometry

## Abstract

**Background:**

Tauopathies represent a diverse group of neurodegenerative disorders characterized by the abnormal aggregation of the microtubule-associated protein tau. Despite extensive research, the mechanisms underlying the diversity of neuronal and glial tau pathology in different tauopathies are poorly understood. While there is a growing understanding of tauopathy-specific differences in tau isoforms and fibrillar structures, the specific composition of heterogenous tau lesions remains unknown. Here we study the protein composition of tau aggregates in four major tauopathies: Alzheimer's disease (AD), corticobasal degeneration (CBD), Pick's disease (PiD), and progressive supranuclear palsy (PSP).

**Methods:**

We developed an approach for in situ proximity labeling and isolation of aggregate-associated proteins using glass slides with formalin-fixed paraffin-embedded (FFPE) human postmortem brain tissue, termed Probe-dependent Proximity Profiling (ProPPr). We used ProPPr for the analysis of proteomes associated with AT8-positive cellular lesions from frontal cortices. Isolated proximity proteomes were analyzed by data-independent acquisition mass spectrometry. Co-immunofluorescence staining and quantitative data analysis for selected proteins in human brain tissue was performed to further investigate associations with diverse tau pathologies.

**Results:**

Proteomics data analysis identified numerous common and tauopathy-specific proteins associated with phospho-tau aggregates. Extensive validations of candidates through quantitative immunofluorescence imaging of distinct aggregates across disease cases demonstrate successful implementation of ProPPr for unbiased discovery of aggregate-associated proteins in in human brain tissue. Our results reveal the association of retromer complex component vacuolar protein sorting-associated protein 35 (VPS35) and lysosome-associated membrane glycoprotein 2 (LAMP2) with specific types of phospho-tau lesions in tauopathies. Furthermore, we discovered a disease-specific association of certain proteins with distinct pathological lesions, including glycogen synthase kinase alpha (GSK3α), ferritin light chain (FTL), and the neuropeptide precursor VGF. Notably, the identification of FTL-positive microglia in CBD astrocytic plaques indicate their potential role in the pathogenesis of these lesions.

**Conclusions:**

Our findings demonstrate the suitability of the ProPPr approach in FFPE brain tissue for unbiased discovery of local proteomes that provide valuable insights into the underlying proteomic landscape of tauopathies, shedding light on the molecular mechanisms underlying tau pathology. This first comprehensive characterization of tau-associated proteomes in a range of distinct tauopathies enhances our understanding of disease heterogeneity and mechanisms, informing strategies for the development of diagnostic biomarkers and targeted therapies.

**Supplementary Information:**

The online version contains supplementary material available at 10.1186/s13024-025-00817-0.

## Background

Tau protein, encoded by the microtubule-associated protein tau gene *(MAPT)* located on chromosome 17, plays a role in regulating organization and dynamics of microtubules [1, 2]. Abnormal accumulation of insoluble tau aggregates in neurons and glia is a major neuropathologic feature of a group of neurodegenerative disorders with distinct clinical [3, 4], biochemical [5] and neuropathologic features [6], which have been termed tauopathies [7]. Under pathological conditions, tau dissociates from microtubules and undergoes post-translational modifications and aberrant phase transition from soluble intrinsically disordered proteins into filamentous aggregates [8–10]. Clinicopathologic and genetic studies have provided substantial evidence that age-at-onset, disease duration, and severity of antemortem cognitive decline are driven by the burden of tau lesions [11–24]. In addition, over 60 genetic mutations in the *MAPT* gene locus have been linked to tauopathies as either disease-causing or risk factors [25–27]. These findings highlight the critical importance of dissecting the composition of disease-specific and shared tau-associated proteins for the development of therapeutic strategies that target mechanisms of tau aggregation, as well as development of biomarkers for differential diagnosis and disease-specific early detection of tauopathies [28–34].

**Fig. 1:**
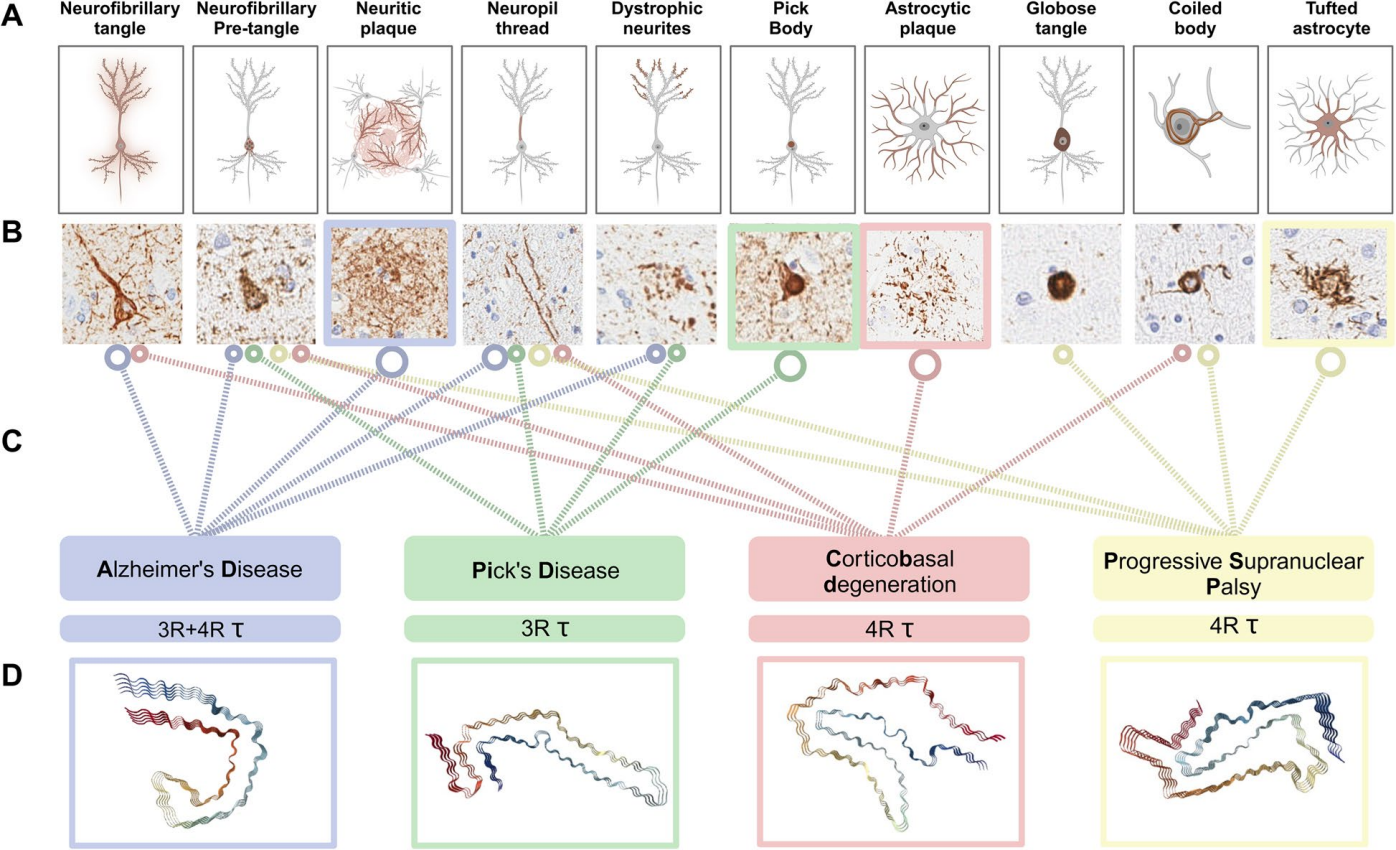
Neuropathological and structure-based characteristics of major tauopathies used for in situ Probe-Dependent Proximity Proteomic profiling of the phospho-tau associated proteome. **A** Schematic depicting the cellular localization of distinct and shared phospho-tau aggregates across Alzheimer’s disease (AD), Pick’s disease (PID), corticobasal degeneration (CBD) and progressive supranuclear palsy (PSP). Hallmarks of AD-related tauopathy include neurofibrillary tangles (NFTs), neuritic plaques (NPs) and neuropil threads (NTs) [49]. PiD is defined by its unique accumulation of spherical intraneuronal argyrophilic inclusions of Pick bodies (PBs), accompanied by dystrophic neurites, NTs and NFTs [50, 51]. PSP is histopathologically diagnosed based on the exclusive presence of tufted astrocyte (TA) lesions in pre-defined brain areas [52], accompanied by globose (GLO-NFT) and premature neurofibrillary tangles, oligodendroglial coiled bodies (CBs) and NTs [53, 54]. CBD shares a clinicopathological spectrum with PSP [55], yet is characterized by the distinctive presence of astrocytic plaques (AP), alongside CBs, NFTs and NTs [56]. **B** Representative human neuropathology images of AT8 stained human brain tissue. **C** Lines indicating the presence of each type of neuropathological lesion in different tauopathies, size of circular end symbol denoting the presence of a defining pathological hallmark specific to either mixed 3R and 4R-, 3R or 4R tauopathies. **D** CryoEM filament structure images generated from RCSB Protein Database (PDB) using the NGL viewer [57] of PDB-ID 503L (AD [41], PDB DOI:https://doi.org/10.2210/pdb5O3L/pdb), PDB-ID 6GX5 (PiD [38], PDB DOI:https://doi.org/10.2210/pdb6GX5/pdb), PDB-ID 6JTO (CBD [43] PDB DOI:https://doi.org/10.2210/pdb6TJO/pdb) and PDB-ID 7P65 (PSP [42] PDB DOI:https://doi.org/10.2210/pdb7P65/pdb). Created with BioRender.com

Currently, over twenty distinct disorders have been classified as either primary or secondary tauopathies [7, 35], depending on whether tau aggregates are the primary disease-causing process or tau is part of a multi-proteinopathy [36]. The neuropathological classification of tauopathies is based on the characteristic features of lesions and their distribution in specific brain regions [6, 9]. This classification is supported by several lines of biochemical evidence. In addition to alternative splice forms in N-terminal exons 2 and 3, the *MAPT* gene produces tau splicing isoforms that contain either 3 or 4 conserved 31–32 amino acid repeats in the microtubule-binding domains [8, 37]. Hence, tauopathies can be classified as either 3R (3-repeat) or 4R (4-repeat) tauopathies or 3R + 4R tauopathies. Primary tauopathies containing tau aggregates formed by 4R tau isoforms include corticobasal degeneration (CBD) and progressive supranuclear palsy (PSP), 3R tau isoforms are primarily found in Pick’s disease (PiD), whereas Alzheimer’s disease (AD) and primary age-related tauopathy (PART) are mixed 3R + 4R tauopathies [6] (Fig. 1). In addition, cryogenic electron microscopy (cryo-EM) studies led to the discovery of a disease-specific taxonomy of tau filament structures [38–44] (Fig. 1). The ability of tau aggregates to differentially seed formation of structurally analogous inclusions in recipient cells suggests that the heterogenous clinical and pathological features of tauopathies may be defined by distinct tau “strains” [44–48]. Disease-specific morphologic and biochemical diversity of tau aggregates has not been studied with unbiased methods to identify common and disease-specific tau associated proteins.

Several studies based on proximity proteomics and immunoprecipitation have aimed to identify tau-associated proteins using a variety of cellular and animal model systems [58]. In addition, the proteome of neurofibrillary tangles (NFTs) from AD brain tissue has been characterized with laser-capture microdissection (LCM) [59]. However, the proteomes associated with the entire spectrum of distinct tau lesions have not been systematically assessed across different diseases, at least in part because of the limitations of available methods for studying local proteomes. Specifically, co-immunoprecipitation is not suitable for discovery of interactomes in insoluble aggregates, due to its sensitivity to the strength of the buffers used during isolation. On the other hand, LCM is not capable of dissection of highly abundant, minute or compartmentally complex lesions, such as neuropil threads (NTs) or astrocytic plaques (APs). Furthermore, due to limitations of current animal models [60, 61], the analysis of morphologically well-preserved pathology from human brain, exhibiting disease relevant filament structures of tau isoforms, with cell type specific and region-dependent vulnerability, is required to identify protein interactors and disease modifiers that are most relevant for human disease. To address these limitations, several in situ proximity proteomics methods based on rapid biotin-tyramide labeling of proximal proteins via horseradish peroxidase (HRP) conjugated antibodies, have been developed for a variety of different applications. This approach has been named enzyme-mediated activation of radical sources (EMARS) [62, 63], selective proteomic proximity labeling assay using tyramide (SPPLAT) [64, 65] or biotinylation by antibody recognition (BAR) [66]. The protein labelling radius for HRP-catalyzed proximity biotinylation has been reported in the range between 250–500 nm [67]. Variations of these methods have been used to profile the proteome of alpha-synuclein aggregates in Parkinson’s disease and Lewy body dementia [68]. More recently, a proof-of-concept workflow was used to identify the phospho-tau associated proteome in PSP, although this study did not follow up on proteomics findings with immunohistochemistry (IHC) [69]. Here, we developed a similar strategy that was optimized for formalin-fixed paraffin-embedded (FFPE) tissue sections mounted on glass slides, which we named Probe-dependent Proximity Profiling (ProPPr). Differences in our independently developed protocol include tissue type and preparation, slice thickness, labeling, lysis, labeling, washing, and elution conditions. Using 5 μm thick FFPE is likely to improve tissue penetration of the primary and secondary antibodies and labeling reagents, the general availability of standard glass slide-mounted 5–10 μm sections routinely provided by brain banks makes the method widely applicable, und avoids quickly depleting paraffin blocks when using 10 × thicker vibratome sections. The term ProPPr term reflects its potential broad application for different target-specific probes (e.g., antibodies, aptamers etc.) and to label proximal molecules (proteins or nucleic acids). In the present study, we utilized ProPPr with the AT8 monoclonal antibody (phospho-tau pS202/pT205) as a specific probe for the unbiased profiling of associated proteomes across the 4 major tauopathies (AD, CBD, PiD and PSP). The goal was to identify and quantify both shared and disease-specific proteins associated with tau aggregates distinct to each tauopathy. Subsequently, a set of proteins was selected for validation using double-label IHC in brain tissue of the various tauopathies. These methods discovered distinct patterns of molecular co-localization of specific subsets of tau lesions, highlighting their involvement in the pathogenesis of tauopathies and their potential as therapeutic targets and biomarkers.

## Methods

### Human post-mortem case selection and neuropathologic evaluation

A selection of cases was made from brains donated for diagnosis and research to the Mayo Clinic Florida Brain Bank (IRB: 15–009452) for Alzheimer’s disease, dementia with Lewy bodies, progressive supranuclear palsy and related neuropathology disorders. Informed consent was obtained from the legal next-of-kin or someone with authority to grant permission for brain donation. The samples used for research were de-identified. Use of deidentified human samples for research purposes is approved by the Biospecimen Committee of the Mayo Clinic Institutional Review Board. Key demographic and clinical data, which were derived from review of available medical records, were used to select cases and their linked demographic and pathologic findings. Age at onset (AAO) was estimated as the time that either the patient or caregiver first reported evidence of impairment. Disease duration was calculated as the interval period AAO and age at death. Sex and ethnoracial status were self-reported by patients or their next-of-kin. Brains were fixed in 10% neutral buffered formalin followed by systematic sampling of tissue after gross neuroanatomical examination. Estimated whole brain weight was calculated by doubling weight of the fixed hemibrain. Cortical sections were sampled perpendicular to the gyrus to maintain integrity of the full cortical ribbon. Blocked tissue samples were embedded in paraffin wax with an automated tissue processor and manual embedding of the permeated tissue in paraffin blocks. Histological assessment for post-mortem diagnosis was conducted by a single expert neuropathologist (D.W.D.), following standardized neuropathologic methods for characterization of cases, as previously described [70]. Briefly, cases were examined according to established guidelines for the criteria to satisfy the diagnosis of intermediate to high likelihood of AD using NIA-AA [71–74], frontotemporal lobar degeneration (FTLD)-tau [52, 75–77] or PART [78]. Cerebrovascular disease [3, 79–81], alpha-synuclein [82, 83], and TAR DNA-binding protein 43 (TDP-43) pathology [84–88] were also examined to denote the presence of any concomitant neuropathologic change, in accordance with recognized guidelines.

Tissue blocks from frontal cortex (middle frontal gyrus, Brodmann area 9 (BA9)) were used for ProPPr labeling. The choice of this brain area met the requirements for study design by being a multimodal association cortex that is affected in all tauopathies included in this study and an area with disease-specific tau pathology. It is also a region with relative homogeneity within disease groups and an area with mixed neuronal and glial pathologies. The tauopathies included in this study included four major subtypes – 4R (PSP and CBD), 3R (PiD) and 3R + 4R (AD). AD cases were selected to have advanced pathology, including involvement of the middle frontal gyrus. Specifically, AD cases had Braak NFT stage > IV [89, 90], Thal amyloid phase > 4 [91], and high probability AD according to the National Institute on Aging (NIA)-Alzheimer’s Association and NIA-Reagan criteria [71–74, 92]. PSP and CBD cases were identified according to Rainwater PSP Criteria and published FTLD-tau criteria [52, 75–77]. The presence of cerebrovascular disease was evaluated using a 10-point modified Kalaria score [80], and cases were excluded based on a Kalaria score ≥ 4 for the cortex and basal ganglia regions assessed. Cases with significant coexisting neuropathologic changes, including hippocampal sclerosis [93], presence of TDP-43 pathology [84–88] or α-synuclein pathology [82, 83] in the neocortex were also excluded. The same criteria were applied to aged controls, which had mild aging associated processes, such as PART. These cases had little to no AD pathology in BA9 (Braak stage ≤ III, Thal phase < 1), had no other co-pathologies [80, 86, 87, 93]. They lacked cognitive decline in life [78]. Demographic and clinicopathologic data of cases and controls are provided in Table 1.

**Table 1:** Demographics of cases used for proteomics and candidate validation in medial frontal cortex tissue

Case	Clin.Dx	Path.Dx	Sex	ER	AAO	AAD	DD	BS	TP	CERAD	ABC	NIH.R	APOE	EXP	%TB
1	AD	AD	M	W	67	73	6	VI	5	Frequent	A3B3C3	High	33	P	8.9
2	AD	AD	F	W	64	87	23	V	3	Moderate	A2B3C2	High	NA	P	4.7
3	AD v. VaD	AD	M	W	66	73	7	V	5	Moderate	A3B3C2	High	NA	P	5.1
4	EOAD	AD	F	W	48	58	10	VI	5	Frequent	A3B3C3	High	NA	P	18.7
5	AD	AD	F	W	81	83	2	VI	5	Moderate	A2B3C2	High	NA	P	1.1
6	AD	AD	M	W	69	77	8	VI	5	Moderate	A2B3C2	High	44	P + V	4.6
7	AD v. FTD	AD	M	W	62	73	11	V	4	Moderate	A3B3C2	High	NA	V	NA
8	AD	AD	F	W	60	86	26	V	5	Moderate	A3B3C2	High	NA	V	NA
9	FTD (SD)	PiD	M	W	62	74	12	0	0	None	A0B0C0	Not	33	P	2.9
10	FTD	PiD	F	H	59	68	9	II	2	None	A1B1C0	Low	33	P	2.1
11	FTD	PiD	F	W	61	68	7	I	0	None	A0B1C0	Not	33	P	3.4
12	FTDP	PiD	M	H	65	81	16	I	0	None	A0B1C0	Not	33	P + V	5.3
13	FTD/PPA	PiD	F	W	47	61	14	0	0	None	A0B0C0	Not	NA	V	NA
14	FTD	PiD	M	W	67	75	8	IV	3	None	A2B2C0	Intermediate	NA	V	NA
15	PNFA	CBD	M	W	68	70	2	II	1	None	A1B1C0	Low	34	P	8.3
16	FAD v. FTD	CBD	M	W	60	70	10	0	0	None	A0B0C0	Not	33	P	4.8
17	PSP v. FTD	CBD	F	H	48	55	7	0	0	None	A0B0C0	Not	34	P	5.5
18	AD	CBD	F	W	70	78	8	III	0	None	A0B2C0	Not	33	P	9.7
19	CBD	CBD	F	W	61	68	7	II	2	None	A1B1C0	Low	34	P	4.5
20	AD v. VaD	CBD	F	W	86	89	3	III	2	None	A1B2C0	Not	NA	P + V	3.8
21	PPA	CBD	M	W	50	55	5	0	0	None	A0B0C0	Not	33	V	NA
22	CBD	CBD	F	W	64	67	3	I	0	None	A0B1C0	Not	NA	V	NA
23	PSP	PSP	M	W	59	66	7	I	1	None	A1B1C0	Low	34	P	0.4
24	PPA v. PSP	PSP	F	W	66	71	5	0	2	None	A1B0C0	Low	33	P + V	0.3
25	PSP/AGD	PSP	F	W	55	61	6	III	0	None	A0B2C0	Not	NA	V	NA
26	PSP	PSP	F	H	55	62	7	0	0	None	A0B0C0	Not	NA	V	0.1
27	NA	PART	M	H	NA	78	NA	II	0	None	A0B1C0	Low	33	P	0.0
28	NA	PART	F	W	NA	92	NA	III	1	None	A1B2C0	Not	33	P	0.0
29	NA	PART	M	H	NA	88	NA	III	0	None	A0B2C0	Not	33	P	0.0
30	NA	PART	M	W	NA	89	NA	III	0	None	A0B2C0	Not	23	P	0.0
31	NA	PART	M	W	NA	74	NA	III	0	None	A0B2C0	Not	NA	P	0.0
32	NA	PART	F	W	NA	94	NA	I	2	None	A1B1C0	Low	33	P + V	0.0
33	NA	CTL	M	W	NA	68	NA	I	0	None	A0B1C0	Low	NA	V	NA
34	NA	CTL	F	W	NA	97	NA	II	2	None	A1B1C0	Low	NA	V	NA
35	NA	CTL	F	W	NA	24	NA	0	0	None	A3B3C3	Not	NA	V	NA

*Abbreviations: AAO* age at onset, *AAD* age at death, *ABC* Amyloid Thal phase, tau Braak Stage, neuritic plaque score (CERAD), *AGD* argyrophilic grain disease, *AD* Alzheimer’s disease, *APOE* apolipoprotein E alleles, *BS* Braak stage, *CERAD* consortium to establish a registry of Alzheimer’s disease- neuritic plaque score, *CBD* corticobasal degeneration, *Clin. Dx* clinical diagnosis, *CTL* unaffected control case, *DD* disease duration, *ER* ethno-racial status, *EOAD* early onset Alzheimer’s disease, *EXP* experimental use of case, *FAD* familial Alzheimer’s disease, *FTD* frontotemporal dementia, *NIH.R* National Institute of Health Reagan Score, *P* ProPPr proteomics use, *Path. Dx* primary pathological diagnosis, *PART* primary age related tauopathy, *PiD* Pick’s disease, *PPA* primary progressive aphasia, *sAD* sporadic *AD, SD* semantic dementia, *TP* Thal Phase, *V* Validation of candidate protein case use, *VaD* vascular dementia, *M* male, *F* female, *NA* not applicable, *%TB* AT8 phospho-tau burden percent coverage in medial frontal cortex tissue used for ProPPr experiments

### Quantitative digital pathology

**Fig. 2:**
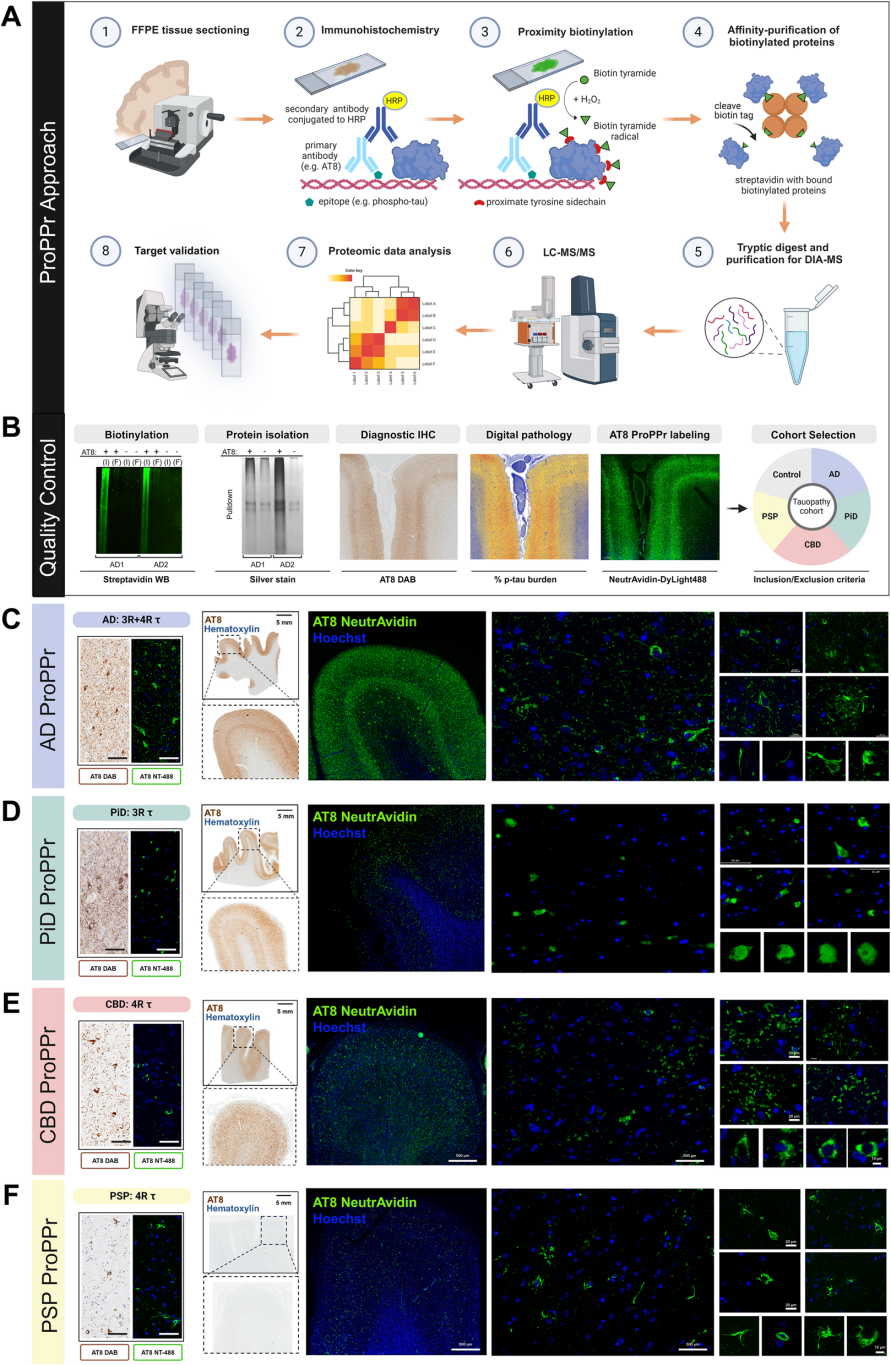
Validation of Probe-dependent Proximity Profiling (ProPPr) method. **A** Schematic representation of the ProPPr method. **B** Western blotting of the tissue lysates reveals extensive biotinylation in the AD samples treated by ProPPr method, but not in the negative controls where staining with AT8 antibodies was omitted. I – input lysate; F – flow-through after pulldown with streptavidin beads. Silver staining of the proteins eluted from streptavidin beads after pulldown revealed increased level of protein in the ProPPr samples comparing to negative controls where staining with AT8 antibodies was omitted. AT8 ProPPr quality control images compared distribution, tau burden by digital pathology and NeutrAvidin-488 labeling of AD frontal cortex. **C-F** Representative images of routine AT8 IHC with and fluorescent neutravidin labeling of proximity biotinylated proteins with AT8 ProPPr in adjacent sections from AD (**C**), PiD (**D**), CBD (**E**), and PSP (**F**) cases. Staining patterns on adjacent sections are evenly matched between the IHC and proximity-biotinylation of phospho-tau in macroscopic cortical tile images and high magnification microscopy demonstrates the specific labeling of tau lesions found in each tauopathy. Created with BioRender.com

For each case used for ProPPr, phospho-tau was assessed with quantitative digital pathology. Glass-mounted 5-µm thick sections from FFPE tissue were processed on a Thermo Fisher Lab Vision 480S autostainer for IHC staining using phospho-tau antibody (AT8 (pS202/T205), 1:1000, Thermo Fisher Scientific, MN1020) and diaminobenzidine (DAB) detection with hematoxylin counterstain. Digital pathology methods using Aperio slide scanners (Leica) have previously been described [3, 94]. In brief, AT8 and hematoxylin counter-stained slides were digitized using the Aperio AT2 scanner. Subsequently, image files were traced and annotated using Aperio ImageScope software (Leica Biosystems, version 12.4.3.5008). Batch analysis was conducted in eSlideManager (Leica), where color deconvolution and custom macros were used to measure phospho-tau burden on AT8 stained sections to produce quantitative pathology data for each case (% tau burden) (Table 1). Representative high-resolution images were acquired at 2 × and 20 × using ImageScope and exported for illustrative purposes (Figs. 1 and 2).

### Phospho-tau proximity labeling for ProPPr

Phospho-tau proximity labeling was performed using 5-µm thick FFPE sections of frontal cortex. For each case, a total of 13 consecutive sections were used, including one section assigned for fluorescence staining to establish specific and efficient proximity labelling. The remaining 12 sections were assigned for either AT8-mediated proximity labelling or negative control (omitting primary antibody) (6 consecutive sections each). Tissue was deparaffinized by immersion in xylene and rehydrated through a series of graded ethanol solutions (100%, 90% and 70% ethanol). Endogenous peroxidase activity was quenched using immersion in absolute methanol containing 0.3% H_2_O_2_ (Sigma-Aldrich, H1009) for 20 min. Antigen retrieval using heated citrate buffer, pH 6 (Dako Target Retrieval Solution, S2369) was performed in a steamer for 30 min. Tissue sections in solution were cooled to room temperature (RT) and washed with a continuous flow of dH_2_O for 10 min. Sections were subsequently blocked and permeabilized using 10% normal goat serum (NGS, Sigma-Aldrich, G9023) in Tris-buffered saline (TBS, DAKO, S196830), containing 0.3% Triton-X 100, at RT for 1 h. Tissues were then incubated with AT8 phospho-tau antibody (1:500, Thermo Fisher Scientific, MN1020) in antibody diluent (5% NGS with 0.3% Triton X-100 in TBS) overnight at 4 °C in a humidified chamber. Tissue sections assigned to negative control samples were incubated overnight in antibody diluent without adding primary antibodies. Tissue sections were washed three times (5 min each) in TBS containing 0.05% Tween-20 (TBS-T). Antibody diluent containing secondary antibody (1:500, F(ab’)2 goat anti-mouse cross-adsorbed HRP, Invitrogen, A24524) was incubated at RT for 1 h, followed by three 5 min TBS-T washes. Immunostained sections were washed three times (5 min each) with Wash Buffer followed by HRP-mediated proximity biotinylation in Labeling Buffer (1xTBS, 5 µM biotin-ss-tyramide (Iris Biotech, LS-3570), 0.03% H_2_O_2_) for 5 min. The reaction was stopped by a 5-min incubation of slides with Quenching Buffer (1xTBS, 0.5 M sodium ascorbate).

One section from each case was used for fluorescent labeling of proximity-biotinylated proteins as a quality control. Sections were washed three times (5 min each) with TBS-T and incubated with NeutrAvidin-DyLight-488 (diluted in antibody diluent, 1:500, Invitrogen, 22,832) for 1 h at RT. Additional sections were also incubated with NeutrAvidin-DyLight-650 (1:500, Invitrogen, 84,607) together with goat anti-mouse Alexa Fluor-488-conjugated antibody (1:1000, Invitrogen, A32723) to evaluate the precision and specificity of proximity biotinylation by co-localization analysis. Tissue was washed with TBS-T and incubated with Hoechst 33,342 (1:1000, Thermo Fisher Scientific, 62,249) for 20 min at before rinsing with TBS. To quench autofluorescence, tissues were stained with 1 × TrueBlack lipofuscin autofluorescence quencher (Biotium, 23,007) for 30 s, rinsed with dH_2_O and mounted with glass coverslips using ProLong Glass Antifade Mountant (Invitrogen, P36980).

### Affinity purification of biotinylated proteins for ProPPr

After proximity labeling, antibodies were stripped from the tissue sections by incubation with 25 mM glycine, pH 2.0 for 30 min. Tissue was then scraped from the glass slides with a razor blade and placed into 0.5 mL Precellys tubes supplemented with CK14 beads (Bertin Technologies, P000933-LYSK0-A) for homogenization. For each case, 6 proximity labeled or 6 control sections were combined into a single tube and supplemented with 200 µl Lysis Buffer I (50 mM ammonium bicarbonate, 0.2% RapiGest SF (Waters)). Tissue was homogenized using a Precellys 24 instrument (Bertin Technologies) by applying two 30 s cycles at 6500 rpm each with 30 s intervals between cycles. Homogenates were transferred to 0.2 µl PCR tubes and incubated at 95 °C for 30 min. Lysates were sonicated for 30 s at 40% amplitude using a Qsonica Q125 sonicator and further incubated at 80 °C for 2 h. Lysates were cleared by centrifugation at 21,000 x*g* for 15 min. Supernatants were collected in clean 1.5 ml Protein LoBind Tubes (Eppendorf), and pellets were resuspended in 200 µL Lysis Buffer II (50 mM ammonium bicarbonate, 8 M urea), incubated for 15 min at RT and centrifuged for 15 min at 21,000 xg*.* The supernatant was mixed with the cleared lysate from previous centrifugation step, and a 5% aliquot from the resulting samples was retained for further mass spectrometry analysis of total lysate. The remaining sample was incubated with 20 µl Streptavidin Sepharose High Performance beads (Cytiva, 17,511,301) overnight with rotation, at 4 °C. Beads were washed 2 times in Wash Buffer I (50 mM ammonium bicarbonate, 0.2% Zwittergent 3–16) and 2 times in Wash Buffer II (50 mM ammonium bicarbonate, 6 M urea). Biotinylated proteins were eluted from the beads by reducing the disulfide bond in the linker between biotin and tyramide using the Elution Buffer (50 mM ammonium bicarbonate, 6 M urea, 10 mM TCEP) with two sequential 15 min incubations at 37 °C.

### Western Blotting and Silver Staining of Protein Gels

For Western blot analysis, 5% of total FFPE tissue lysates were mixed with 4 × Bolt LDS Sample Buffer (B0008, Invitrogen) without adding any reducing agents. The samples were heated at 95 °C for 10 min before loading onto Bolt 4–12% gradient Bis–Tris polyacrylamide gels. Electrophoresis was conducted in Bolt 1 × MES Running Buffer (B0002, Invitrogen) at a constant voltage of 5 V/cm.

Following electrophoresis, total lysate samples were transferred to nitrocellulose membranes using the iBlot 3 Dry Blotting System (Thermo Fisher Scientific). After protein transfer, the membranes were dried for 10 min at 37 °C and stained using the Revert 700 Total Protein Staining Kit (926–11,010, LI-COR) for normalization, according to the manufacturer’s protocol. Membranes were scanned using the Odyssey DLx Imaging System (LI-COR) and subsequently de-stained with the Revert 700 destaining solution (926–11,023, LI-COR). The membranes were then blocked with Intercept PBS Blocking Buffer (927–70,001, LI-COR) for 1 h at room temperature and incubated overnight at 4 °C with IRDye 800CW-conjugated streptavidin (1:10,000, 926–32,230, LI-COR). After three washes in PBS-T (1 × PBS, 0.1% Tween 20), the membranes were scanned again using the Odyssey DLx Imaging System. Quantification of fluorescent signals from total protein staining and Western blotting was performed using Image Studio Lite Software (version 5.2, LI-COR). Subsequent data analysis and plot generation were conducted in the RStudio development environment (version 2023.06.1, Posit) for R (version 4.3.1).

For silver staining, 10% of the eluates from the Streptavidin Sepharose beads were processed and separated by electrophoresis as described above. The gels were then stained using the Pierce Silver Stain Kit (24,612, Thermo Fisher Scientific) according to the manufacturer’s instructions. Images of the stained gels were captured using the ChemiDoc MP Imaging System (Bio-Rad).

### Mass spectrometry

Total lysate samples were reduced with 10 mM tris(2-carboxyethyl)phosphine (TCEP) in 50 mM ammonium bicarbonate buffer for 30 min at 37 °C and alkylated with 20 mM solution of iodoacetamide in 50 mM ammonium bicarbonate buffer for 30 min at room temperature in the dark. Proteins eluted from the beads of pull-down samples were alkylated without reduction. Proteins were then incubated with Lys-C/trypsin mixture (Promega, V5073) (200 ng for pull-down samples and 1 µg for total lysate samples) for 2 h at 37 °C. Afterwards the samples were diluted to decrease the maximum concentration of urea (up to 1 M) and supplemented with additional amounts of sequencing-grade trypsin (Promega, V5111) (200 ng for pull-down samples and 1 ug for total lysate samples), followed by overnight incubation of the samples at 37 °C. Before mass spectrometry analysis samples were de-identified and randomized. Peptides were directly loaded on an Evosep C18 tip and separated using the Evosep One with the 100 spectral power distribution (SPD) High Organic method (Evosep, Odense, Denmark). Peptides were eluted and ionized using a Bruker Captive Spray emitter. A Bruker timsTOF Pro 2 mass spectrometer running in diaPASEF (parallel accumulation – serial fragmentation combined with data-independent acquisition) mode [95] was used for data acquisition with 6 × 3 50 m/z windows per PASEF scan.

### Proteomic data analysis

Data-independent acquisition (DIA) mass spectrometry data were analyzed with the Spectronaut 17 software package (Biognosys) using the targeted library-based approach. A spectral library was generated by searching the data for both pull-down and total lysate samples using the Spectronaut pulsar search engine with the default setting against the Uniprot UP000005640 Human database (20,607 entries) supplemented with the common contaminants database (38 entries). Pulldown samples were separately matched with the resulting library using the Spectronaut 17 default settings. Briefly, “trypsin/P specific” was selected to allow for two missed cleavages. Fixed modifications were set to cysteine carbamidomethylation, and variable modifications were set to peptide N-terminal acetylation and methionine oxidation. For DIA search parameters, PSM and Protein Group decoy false discovery was set to 1%. Protein level intensities were summarized using the MaxLFQ algorithm. Phospho-tau associated proteins were identified using the MiST computational tool that was originally developed for scoring affinity purification mass spectrometry data using pull-down samples and their corresponding negative controls for each pathology separately [96]. For the analysis of differential protein association with AT8-positive phospho-tau in different disorders, data normalization, imputation of missing values and differential abundance test was performed using the DEP R package (version 1.22.0) [97]. Proteins were considered significantly different in each of the pairwise comparisons when adjusted *p*-value was less than 0.05. Gene Ontology (GO) term overrepresentation analysis was performed using the clusterProfiler R package (version 4.8.2) [98]. GO term networks were generated by the EnrichmentMap plugin (version 3.1.0) and protein–protein interaction network was imported from the StringDB database using the stringApp plugin (version 2.0.2) in Cytoscape software (version 3.9.1) [99–101]. Venn diagrams were generated using the online tool for calculation and drawing of Venn diagrams developed by the University of Ghent (https://bioinformatics.psb.ugent.be/webtools/Venn/). The statistical significance of the overlap between gene sets was calculated by a hypergeometric test using the online tool from the Graeber lab at the University of California, Los Angeles (https://systems.crump.ucla.edu/hypergeometric/index.php). Plots were created in the RStudio development environment (version 2023.06.1, Posit) for R programming language (version 4.3.1) using the ggplot2 R package [102].

### Immunofluorescence (IF) imaging

Additional 5 µm FFPE frontal cortex tissue sections from select cases were used for validation of candidate protein associations with phospho-tau by co-immunofluorescence tissue staining (Table 1). Slides were treated as described above for proximity labeling using the methods of deparaffinization, antigen retrieval, while omitting endogenous peroxidase quenching, with only immersion in absolute methanol for 20 min to reduce autofluorescence. Following rinsing with dH_2_O for 5 min, tissue sections were simultaneously blocked and permeabilized using serum-free protein block (Dako, S0909) containing 0.3% Triton-X 100 at RT for 1 h. Slides were co-immunostained with AT8 (1:600, Invitrogen, MN1020) and one of the following primary antibodies for selected candidates: vacuolar protein sorting-associated protein 35 (VPS35) (1:400, GeneTex, GTX108058); LAMP2 (1:400, Abcam, ab199946); VGF (1:200, Abcam, ab308287); glycogen synthase kinase 3-alpha (GSK3α) (1:200, Abcam, ab1340870); ferritin light chain (FTL) (1:400, Novus, NBP2-34,072). LAMP2 double immunolabelling with conformational tau antibody (MC1, gift from the late Peter Davies, Feinstein Institute for Medical Research), was coupled with thioflavin S (Thio-S) staining. In some CBD cases, triple immunofluorescent staining was performed using antibodies to FTL, cell type specific markers for astrocytes [GFAP—1:1000 (Invitrogen, 2.2B10)] or microglia [IBA1—1:500 (Abcam, ab300156)] and phospho-tau (AT8) (refer to Supplementary Table 1 for antibody/stain combinations). Tissue sections were incubated with primary antibodies diluted in antibody diluent (Dako, S0809) containing 0.3% Triton X-100 overnight at 4 °C in a humidified chamber. Sections were then washed with TBS-T three times for 10 min each and incubated with Alexa Fluor conjugated secondary antibodies diluted in antibody diluent for 2 h at RT in the dark. For labelling experiments that required fluorescent secondary antibodies for two markers (e.g., AT8 and other candidate proteins), anti-rabbit F(ab’)2-AlexaFluor-488 and anti-mouse F(ab’)2-AlexaFluor-647 secondary antibodies were used to reduce the possibility of spectral crosstalk/bleed-through. Triple fluorescent labeling was conducted using secondary antibody combinations without cross-reactivity (anti-rabbit F(ab’)2-AlexaFluor-488, anti-rat F(ab’)2-AlexaFluor-555 and anti-mouse F(ab’)2-AlexaFluor-647) (Supplementary Table 1). After incubation, tissue sections were washed with TBS-T and incubated with Hoechst 33,342 (1:500, Thermo Fisher Scientific, 62,249), followed by autofluorescence quenching with 1 × True Black (Biotium, 23,007) before mounting (ProLong Glass Antifade mountant, Invitrogen, P36980). Thioflavin S was used to stain specific cases for amyloid found in senile plaques and tangles, after completion of secondary antibody staining (either with GFAP and IBA1 or with LAMP2 and MC1), but before Hoechst staining and lipofuscin pigment quenching. Briefly, tissue was washed with 40% ethanol for 1 min and incubated with 300 µl of Thioflavin S solution (25 mM in 50% ethanol/TBS) for 3 min, followed by three consecutive one-minute washes in 50% ethanol and three rinses in TBS. Afterwards, Hoechst staining, autofluorescence quenching and mounting was performed as described above.

Single field of view (FOV) imaging was conducted using a Keyence BZ-X800 inverted fluorescence microscope. Slides were subject to high resolution tile imaging and high magnification z-stack imaging, using 20x, 60 × or 100 × objectives, respectively. Tile images were captured using a 20x, 40 × or 60 × objectives, and tiles were sequentially mapped with corresponding z-stacks. Image files for each single channel and the merge channel tile scan, from overlapping x–y and 3–5 µm z series, were reconstructed using the XY stitching function without compression before full focus projection to generate the final high resolution projected tile images. Single FOV images for double or triple staining were acquired using sequential z-series and 20x, 60 × or 100 × objectives. Images were acquired using the high-resolution acquisition capture mode through a 60 × or 100 × objective, which permitted single plane images to be projected in full focus from the z-series of sequential optical sections. The depth of each z-stack typically spanned 3–5 µm of the tissue at the point of interest and was dependent on capturing the major aggregates or tissue distribution of candidate proteins/cell types. High-resolution tiled images of FTL, IBA1 or GFAP and AT8, were reconstructed by stitching without compression, and exported using the Keyence analyzer package (BZ-X series). For the quantitative analysis of protein associations with phospho-tau lesions we acquired 5 × 5 FOV tiled images of double immunostained tissue sections using 20 × objective on Keyence BZ-X800 inverted fluorescence microscope.

Tissue sections from tauopathies that were co-stained with AT8 and NeutrAvidin-DyLight-650 were imaged on Eclipse Ti2 fluorescence microscope (Nikon) equipped with a Spectra X multi-LED light engine (Lumencor), single bandpass filter cubes for DAPI, EGFP/FITC and TRITC (Chroma), and a Zyla 4.2 PLUS sCMOS camera (Andor), using NIS Elements HC V5.30 software (Nikon) using 20 × and 100 × objectives.

### Quantitative analysis of protein association with AT8 phospho-tau lesions

For each case, the pertinent pathologies for each disease were analyzed for their relationship with the phospho-tau-associated proteins. Tau neuropathological nomenclature in the current study refers to the following: NFT = neurofibrillary tangle; PT = pre-tangle; TANC = tangle-associated neuritic cluster; GLO-NFT = globose/ballooned neuron in PSP; BN = ballooned neuron in CBD; NT = neuropil thread; NP = neuritic plaque; AP = astrocytic plaque; TA = tufted astrocyte; CB = coiled body; GT = glial tau in PiD, SPL – small phospho-tau lesions in CBD. The quantification was conducted in two or more cases for each disease (see Table 1 for case utility).

Composite multichannel images from co-immunostaining experiments were first pre-processed and analyzed with Fiji distribution of ImageJ software (version 2.14.0) using custom-written macros (Supplementary Fig. 1). The images were first split into single-channel images, followed by signal thresholding and creating binary images for AT8 and associated protein staining. In some cases, thresholding was preceded by background subtraction due to high level of background signal in the images (VGF). Binary images from single-channel data were combined to identify overlapping pixels using the “Image Calculator” plugin in ImageJ. Regions of interest (ROIs) for each image or lesion type were either manually drawn on original multichannel images (NFT, NP, TANC, AP, TA, GLO-NFT, CB, PB, GT and SPL) or detected automatically (NT). Automatic NT detection involved processing binary images from the AT8 channel after manually excluding other ROIs using the “Analyze Particles” function in ImageJ, with filtering criteria based on area (≥ 0.01 μm^2^) and aspect ratio (> 3).

The defined ROIs were overlaid onto the “AT8” and “overlap” binary images. For each ROI, the percentage of the area occupied by positive pixels was measured and recorded as Area_AT8_ and Area_overlap_. The proportion of each lesion overlapping with the studied protein was calculated as Area_overlap_/Area_AT8_. Lesions were categorized based on the overlap proportion as follows: “strongly positive” (overlap ≥ 70%), “moderately positive” (overlap between 50 and 70%), “weakly positive” (overlap between 20 and 50%) and “negative” (overlap < 20%).

### Statistical analysis

Statistical analyses were conducted using R (version 4.4.2) within the RStudio integrated development environment (version 2024.09.0). Correlations between quantitative protein measurements from Western blot and mass spectrometry-based proteomics were assessed using Spearman's rank correlation coefficient. Differences in the frequencies of co-localization categories for phospho-tau lesions and associated proteins of interest were evaluated using pairwise Fisher’s exact tests, with *p*-values adjusted for multiple comparisons using the Bonferroni method. Differences in total area of co-localization between phospho-tau lesions were analyzed using the Kruskal–Wallis test followed by post hoc Dunn’s test. The results of pairwise statistical comparisons are summarized in the Supplementary Fig. 8.

## Results

### Development and validation of ProPPr

For labeling and purification of phospho-tau-associated proteomes from post-mortem human tissue with ProPPr, we utilized the AT8 antibody as a probe for HRP-catalyzed biotinylation of proximate proteins followed by their isolation with streptavidin beads (Fig. 2A). Briefly, phospho-tau aggregates in 5 μm post-mortem FFPE brain tissue were immunostained with AT8 and HRP-conjugated anti-mouse secondary antibodies using standard IHC. HRP catalyzes the local formation of highly reactive biotin-tyramide radicals, leading to the biotinylation of proximal protein tyrosine side chains. Proximity-labeled proteins are then affinity-purified from tissue lysates with streptavidin beads and analyzed by mass spectrometry.

To evaluate the concordance of protein labeling using biotin-ss-tyramide, we performed double fluorescence IHC and ProPPr staining on sections from all four major tauopathies with NeutrAvidin-DyLight 650 and Alexa Fluor 488-conjugated anti-mouse secondary antibodies. The signals from NeutrAvidin and the secondary antibodies were found to overlap, demonstrating that proximity biotinylation is confined to AT8 + areas in close proximity to phospho-tau epitopes (Supplementary Fig. 2A). To further establish the specificity of the ProPPr method, western blot analysis of tissue lysates with fluorescent streptavidin for each case was used to confirm protein biotinylation in AT8 antibody-positive versus negative samples. We observed a strong difference in streptavidin signal between AT8 + and AT8- samples, indicating specific labelling of proteins with biotin-ss-tyramide in tissue sections stained with the AT8 antibody (Supplementary Fig. 2B, 2C). The ratios of normalized biotinylation signals between AT8 + and AT8- samples correlated significantly with tau burden quantified by digital pathology (Spearman’s rho = 0.85, p < 0.001) and number of proteins identified by mass spectrometry (Spearman’s rho = 0.65, p < 0.001) (Supplementary Fig. 2D, 2E), indicating that the results of mass spectrometry analysis reflect the labelling efficiency of tissue section in ProPPr. To assess the amount of protein isolated from these lysates with streptavidin beads, for 2 individual AD cases (case #1 and case #3) proteins were eluted from the beads and separated by SDS-PAGE followed by silver staining of the gel. A significant difference in amounts of affinity-purified protein was observed between AT8-positive ProPPr samples comparted to negative controls with primary antibody omission, indicating the efficiency and specificity of the ProPPr method (Fig. 2B). Further quality controls assessed the spatial distribution of proximity labelled proteins by NeutrAvidin-DyLight 488 staining, closely matching the AT8-DAB IHC staining pattern of adjacent tissue slides from the same cases (Fig. 2B-F). Specifically, the laminar distribution of AT8 pathology in AD cortex, in layers III and V of the neocortex in AD was similarly highlighted by digital neuropathology methods for tau burden quantification. For further validation of the ProPPr approach, we carefully determined that disease-specific tau inclusions were labelled with fluorescent streptavidin, while we did not observe any significant signal in the cognitively unaffected control cases, indicating high specificity of ProPPr proximity biotinylation (Fig. 2C-F, Supplementary Fig. 2F).

### Comprehensive profiling of phospho-tau-associated proteins by ProPPr

**Fig. 3:**
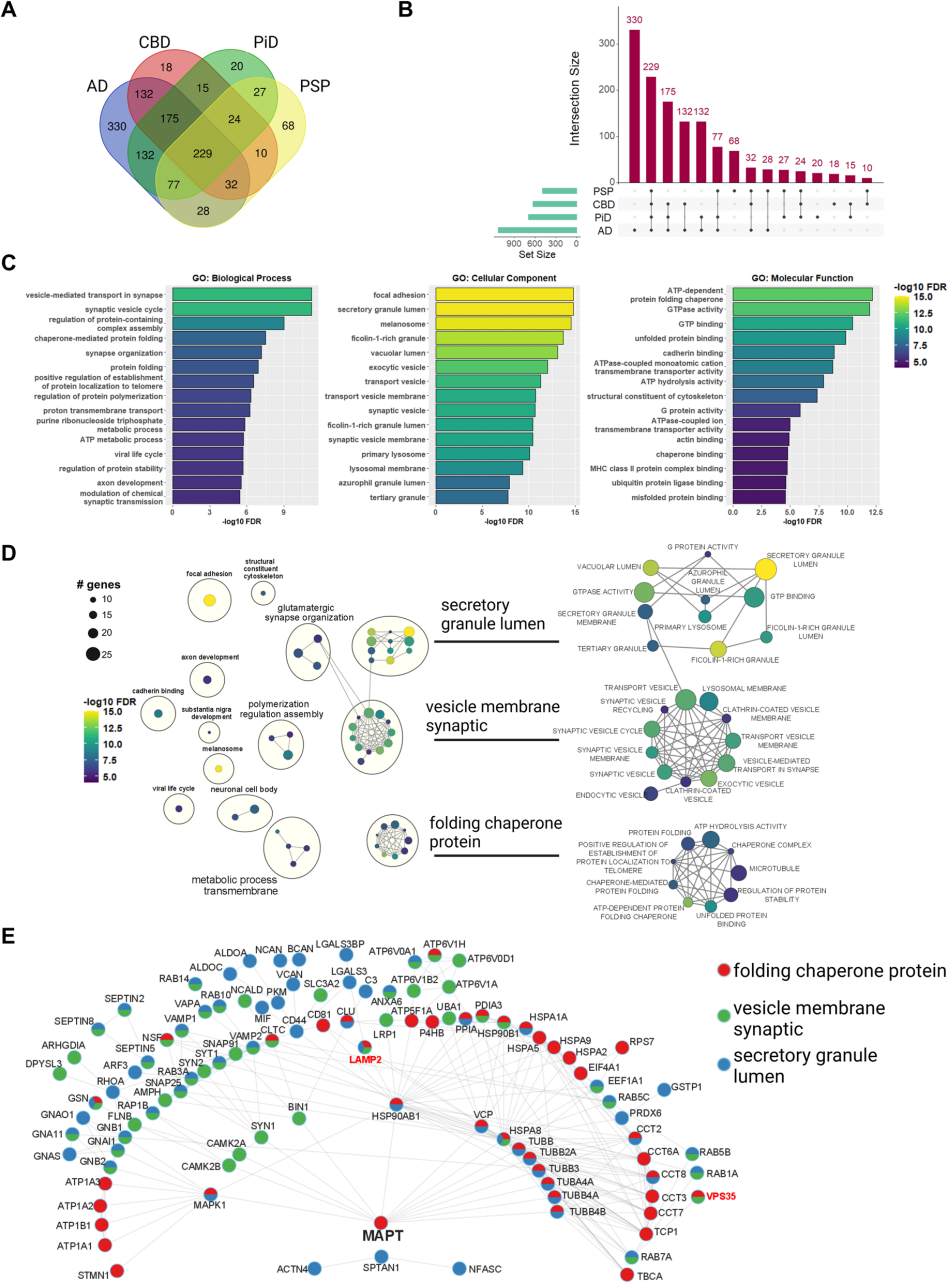
Overview of phospho-tau associated proteome across tauopathies. **A** Venn diagram for overlaps between the identified phospho-tau-associated proteins in tauopathies. **B** Upset plot for overlaps between the identified phospho-tau-associated proteins in tauopathies. **C** Top-15 terms from each GO category enriched in the set of phospho-tau associated proteins identified in all the tauopathies (229 proteins). **D** Enrichment Map network of top-50 GO terms enriched in the set of phospho-tau associated proteins identified in all the tauopathies (229 proteins). Clusters of nodes were annotated with the Autoannotate Cytoscape plugin. Color of the node represents false discovery rate (FDR), size of the node corresponds to the number of genes in GO terms. Right panel contains close-up on the largest clusters of the enriched GO terms. **E** STRING network of protein–protein interactions for proteins from “secretory granule lumen”, “vesicle membrane synaptic” and “folding chaperone protein” clusters of GO terms

The ProPPr method was applied to discover the composition of disease-specific phospho-tau-associated proteomes in post-mortem frontal cortices in four major tauopathies (see Table 1). The association probability score of phospho-tau associated proteins was determined separately for each disease group using Mass spectrometry interaction Statistic (MiST) analysis [96], employing summarized protein intensity values from positive samples and their corresponding antibody-omitting negative controls. The MiST scoring system uses unsupervised machine learning to integrate three measures—abundance, reproducibility, and specificity—calculated from mass spectrometry proteomics data for each bait-prey pair into a composite score ranging from 0 to 1 [96]. Using a MiST score cut-off value of 0.75, we identified 1,317 phospho-tau-associated proteins, corresponding to 1,314 unique protein-coding genes (Supplementary Table 2). Specifically, 1,135 proteins were identified as phospho-tau-associated proteins in AD, 635 proteins in CBD, 699 proteins in PiD, and 495 proteins in PSP (Fig. 3A, B).

To establish concordance of our findings with established tau interactomes, we compared our phospho-tau associated protein dataset with previously reported tau interactors from a comprehensive review by Kavanagh et al., [58], which catalogues 2084 identified physiological or pathological tau interacting proteins from 7 diverse experimental models and human brain studies (2035 unique gene names in total) [58, 59, 103–108]. We observed a strong overlap with 844 of 1314 identified genes (~ 64%) from the tau ProPPr dataset having previously been linked to tau interactome studies, showing a statistically significant enrichment of known tau interactors in our phospho-tau-associated proteomes (*p-*value = 1.45*10^–568^ by hypergeometric test) (Supplementary Fig. 3A). Additionally, concordance analysis with a proteome of NFT from AD by laser-capture microdissection [59], revealed 426 out of 542 reported proteins (~ 78%) were also identified in our dataset, which comprises a statistically significant overlap (*p*-value = 7.64*10^–421^ by hypergeometric test) (Supplementary Fig. 3B). Finally, we compared our protein sets with recently published BAR-MS data on phospho-tau associated proteins in FFPE PSP tissue [69]. This study reported only 117 proteins (116 genes) associated with phospho-tau lesions in PSP, which is considerably lower than the 495 proteins (494 unique gene names) we found using the ProPPr protocol, most likely due to the differences in the methodology, analysis pipeline, and different criteria in the BAR-MS study. Nevertheless, our set of phospho-tau-associated proteins in PSP was significantly enriched in proteins identified in the PSP BAR-MS study (57 overlapping genes, *p*-value = 6.4 *10^–61^ by hypergeometric test) (Supplementary Fig. 3C). Our combined set of 1314 genes contained 102 out of 116 genes identified in the PSP BAR-MS study (*p-*value = 1.76*10^–106^ by hypergeometric test), suggesting that some of the phospho-tau-associated proteins identified in PSP brains may not be specific to PSP, but are common to other tauopathies (Supplementary Fig. 3D). Overall, this analysis affirms the reliability of our ProPPr method in identifying known tau interactors and associated proteins, supporting its utility in profiling aggregate-associated proteomes in human tissues. Importantly, 409 proteins that we identified using ProPPr method were not present in the datasets used for validation, making them potentially novel tau aggregate-associated proteins (Supplementary Fig. 3E).

### Common tau pathology-associated proteins are enriched for endomembrane and chaperone proteins

We first proceeded to analyze the set of 229 proteins that we found associated with phospho-tau across all studied tauopathies and are classified as common interactors, by performing Gene Ontology (GO) term overrepresentation analysis. The most significantly overrepresented GO Biological Process (GO-BP) terms were “vesicle-mediated transport in synapse” and “synaptic vesicle cycle,” indicating significant presence of synaptic proteins in phospho-tau aggregates (Fig. 3C, Supplementary Table 3). The most enriched GO Cellular Component term was “focal adhesion,” followed by terms associated with intracellular membranous organelles such as secretory granules, melanosomes, lysosomes, and other types of vesicles (Fig. 3C, Supplementary Table 4). Overrepresentation analysis of GO Molecular Function (GO-MF) terms highlighted “ATP-dependent protein folding chaperone” as the most significantly enriched term in this protein set, aligning with the known involvement of chaperones in misfolded tau aggregates (Fig. 3C, Supplementary Table 5).

To further characterize the GO terms overrepresented in the shared phospho-tau associated proteome across tauopathies, we built the Enrichment Map network of the top-50 most significantly enriched GO terms [100]. Among the 50 GO terms used for this analysis we identified 3 major clusters of terms. Based on word frequency in GO term descriptions, these clusters were annotated as “secretory granule lumen,” “vesicle membrane synaptic,” and “folding chaperone” (Fig. 3D). The GO terms from these 3 clusters included 127 out of 229 analyzed proteins. For a more detailed overview of protein groups and complexes sequestered into phospho-tau lesions, we obtained the network of protein–protein interactions for these proteins from the STRING database. We found that 102 out of 127 proteins were involved in high stringency protein–protein interactions (STRING combined score >  = 0.7) with other proteins from the analyzed set (Fig. 3E).

The “folding chaperone” cluster contained 9 GO terms associated with protein folding and regulation of protein stability, as well as the terms “microtubule”, “ATP hydrolysis activity”, and “positive regulation of establishment of protein localization to telomere.” Identified proteins belonging to the protein folding terms included a group of heat shock proteins from the HSP70 (HSPA1A, HSPA2, HSPA5 and HSPA8) and HSP90 (HSP90AB1 and HSP90B1) protein families, constituents of the CCT/TriC chaperonin complex (CCT2, CCT3, CCT6A, CCT7, CCT8 and TCP1), the secreted molecular chaperone clusterin (CLU), the ubiquitin-dependent segregase valosin-containing protein VCP, and peptidyl-prolyl cis–trans isomerase PPIA. Another notable group from this cluster of GO terms consisted of microtubule-associated proteins including tau (MAPT), the microtubule destabilizing protein stathmin 1 (STMN1), and several α- and β-tubulins.

In addition, several protein subunits of Na^+^/K^+^ ATPase (ATP1A1, ATP1A2, ATP1A3 and ATP1B1) were present among the proteins from the “ATP hydrolysis activity” GO term of this cluster. The “secretory granule lumen” GO term cluster contained 11 GO terms associated with secretary granules, guanyl nucleotide binding, and primary lysosome. These terms contained various G protein subunits (GNAI1, GNA11, GNAO1, GNAS, GNB1, GNB2), small GTPases (RHOA, ARF3, RAB1A, RAB3A, RAB5B, RAB5C, RAB6B, RAB7A, RAB10 and RAB14), glycolytic enzymes ALDOA, ALDOC and PKM, chondroitin sulfate proteoglycans versican (VCAN), brevican (BCAN), and neurocan (NCAN), as well as galectin-3 (LGALS3) and galectin-3-binding protein (LGALS3BP).

The “vesicle membrane synaptic” cluster contained 12 GO terms associated with transport vesicles, endocytic vesicles, clathrin-coated vesicles, and lysosomal membranes. The latter term included several subunits of vacuolar type proton ATPase required for lysosome acidification (ATP6V0A1, ATP6V0D1, ATP6V1A, ATP6V1B2 and ATP6V1H), lysosome-associated membrane glycoprotein LAMP2, and the retromer complex protein VPS35. The GO terms from these cluster also included numerous proteins involved in synaptic vesicle recycling, such as SYN1, SYN2, SYT1, SNAP25, VAMP1, VAMP2, SNAP91, AMPH, as well as AD-linked protein BIN1 [109]. These findings support a key role of the endo-lysosomal pathways in the pathogenesis of all major tauopathies [110].

### VPS35 and LAMP2 are sequestered into heterogenous phospho-tau aggregates across tauopathies

**Fig. 4:**
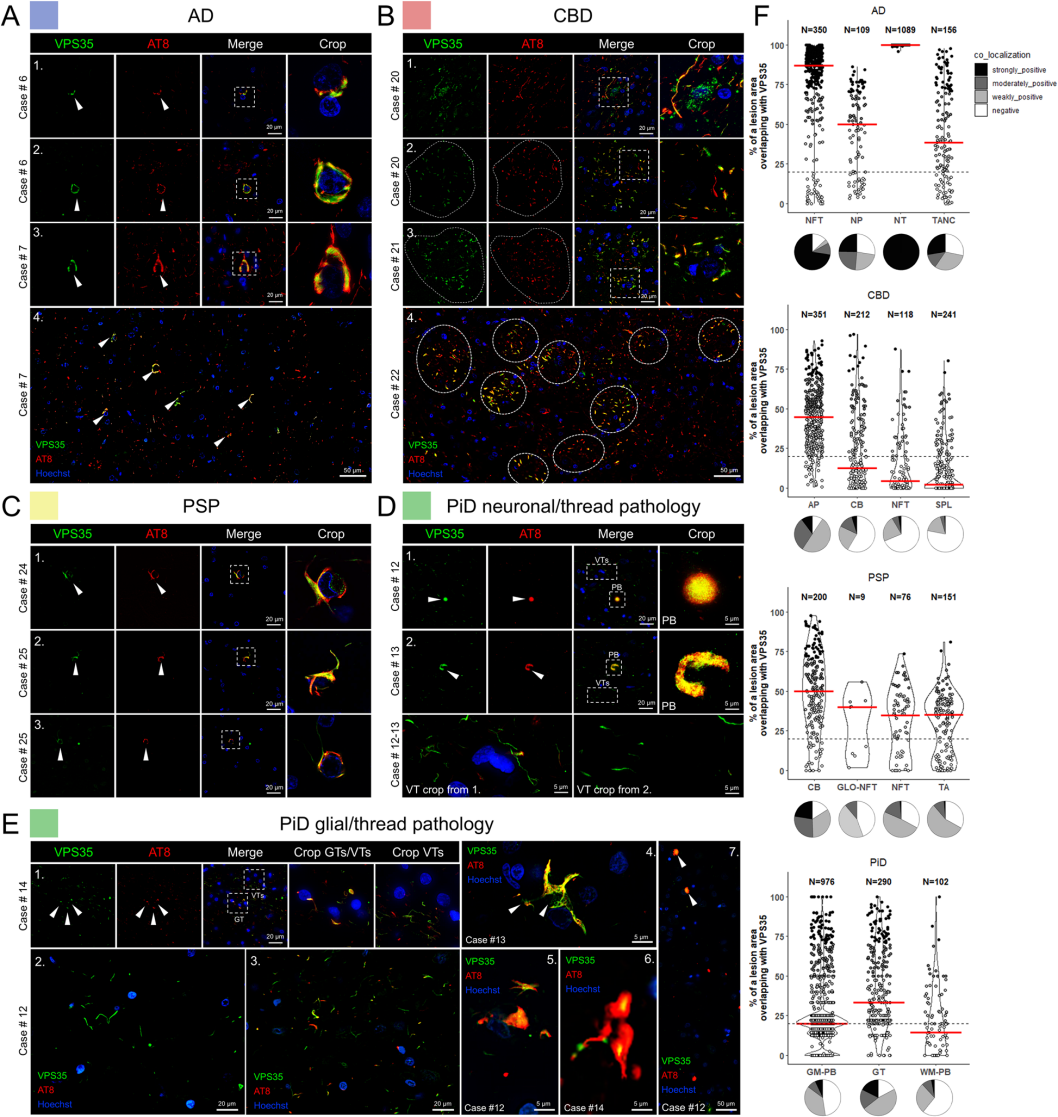
VPS35 co-localizes with specific disease-related AT8-positive tau lesions in post-mortem frontal cortices from major tauopathies. **A** Co-immunostaining of AD brain sections revealed a robust colocalization between VPS35 and phospho-tau (white arrowheads) in neuronal tau lesions, including pre-tangles (series 1), neurofibrillary tangles (NFTs) (series 2), and more mature NFTs (series 3). Co-localization between VPS35 and AT8 phospho-tau is common in NFT-burdened brain regions (series 4). **B** VPS35 is sequestered in APs in CBD brains (series 1–4). Individual APs in 4 are denoted by dashed circles. **C** VPS35 is sequestered in CBs in PSP brains (series 1–3, white arrowheads indicate co-localization). **D** Circumscribed neuronal PBs displayed VPS35 punctate staining within the cores of the tau inclusions (series 1), whereas in larger ‘horseshoe’ shaped neuronal PBs VPS35 puncta were more evenly distributed (series 2, white arrowheads indicate co-localization). In addition, in PiD we observed frequent occurrence of VPS35 thread structures (VTs) that colocalized with AT8 but were mostly AT8-negative (series 1–2, VTs insert). **E** VPS35 is sequestered in glial phospho-tau inclusions (GTs), including oligodendroglial (series 1, Crop GTs/VTs insert) and astrocytic [4–6] phospho-tau inclusions in PiD. Small clusters of VTs were sometimes observed in the vicinity of AT8 oligodendroglial inclusions (series 1, Crop GTs/VTs and Crop VTs inserts). Larger clusters of VTs varied in lengths (series 2). In dense areas of VTs some portion of them was AT8 positive (series 3). Grey matter Pick body inclusions were positive for VPS35 (series 7, white arrowheads), yet at the border of the white matter, smaller spherical inclusions were negative for VPS35. **F** Quantification of VPS35-positive phospho-tau lesions from *n* = 3 cases/tauopathy showing the relative area of overlap between AT8 and VPS35 in tauopathy lesions. Modified violin plots with data points show individual lesions plotted against relative overlap area. Lesions are categorized into co-localization categories based on their relative area of overlap. Red lines indicate median values. Pie charts underneath the violin plots illustrate the relative proportions of lesions with different degrees of co-localization. Abbreviations: VPS35, vacuolar protein sorting-associated protein 35; AD, Alzheimer’s disease; PiD, Pick’s disease; CBD, corticobasal degeneration; PSP, progressive supranuclear palsy; PTs, pre-tangles; NFTs, neurofibrillary tangles; TANCs, tangle-associated neuritic clusters; APs, astrocytic plaques; CBs, coiled bodies; PBs, Pick bodies; VTs, VPS35 threads; GT, glial tau; NTs, neuropil threads; NP, neuritic plaques; SPL, small phospho-tau lesions; TA, tufted astrocytes; GLO-NFT, globose NFTs; GM PB, grey matter Pick Body; WM PB, white matter PB

Among the phospho-tau associated proteins shared across tauopathies, we selected VPS35 and lysosome-associated membrane protein (LAMP2) for further studies (Fig. 3E). VPS35 is a member of the cargo-selective retromer complex, together with VPS26 and VPS29, and constitutes an integral part of functional retromer assembly facilitating endosomal trafficking of cargo-specific transport from endosomes to the trans-Golgi network or plasma membrane [111]. The retromer complex and its components, including VPS35, have been implicated in several neurodegenerative diseases [112]. Most notably, the D620N mutation in the VPS35 protein is causative of late-onset Parkinson’s disease in an autosomal-dominant manner [113]. Given these strong links to neurodegeneration, we decided to explore the association of VPS35 with phospho-tau lesions by co-immunostaining post-mortem frontal cortex sections across tauopathy cases with AT8 and VPS35 antibodies (Fig. 4). In AD, VPS35 demonstrated the most robust co-localization with both AT8 positive pre-tangles, mature NFTs and NTs in all cases examined (Fig. 4A, F, Supplementary Fig. 4A). In CBD frontal cortex, VPS35 exhibited strong co-localization with AP pathology (Fig. 4B, F). APs of differing densities reflect the sequestration of VPS35 that starts from the early phases of AP development and continues during the plaque growth (Fig. 4B). VPS35 was sequestered in the distal extensions of APs and strongly co-localized with a large portion of AT8 phospho-tau throughout the cortical regions of CBD cases with high phospho-tau burden (Supplementary Fig. 4D). In PSP frontal cortices, most of the oligodendroglial CBs were positive for VPS35, while TAs and neuronal GLO-NFTs were co-labeled less frequently (Fig. 4C, F, Supplementary Fig. 4C). In PiD, VPS35 was found in PBs and they had punctate staining within the lesion (Fig. 4D, F, Supplementary Fig. 4B). In the white matter, VPS35 signal was present in smaller AT8 lesions, likely representing glial phospho-tau aggregates (Fig. 4E, F). Intriguingly, in PiD we observed VPS35 immunoreactivity in thread-like structures of varying lengths, which were often negative for phospho-tau or only partially co-localized with AT8 (Fig. 4D, E). This pathology was not observed in cognitively unaffected individuals or in lesions of other tauopathies. Larger heterogeneous glial phospho-tau lesions varied in the degree of VPS35 protein sequestration (Fig. 4E, F). Grey matter PBs (GM-PB) were mostly positive for VPS35, yet smaller spherical possibly glial inclusions at the gray-white junction (WM-PBs), had lower frequencies of high degree co-localization (Fig. 4E, F). All normal control cases that were negative for AT8 had physiologic cytosolic distribution of VPS35 at far lower intensity than of VPS35 in AT8 lesions (Supplementary Fig. 4E).

**Fig. 5:**
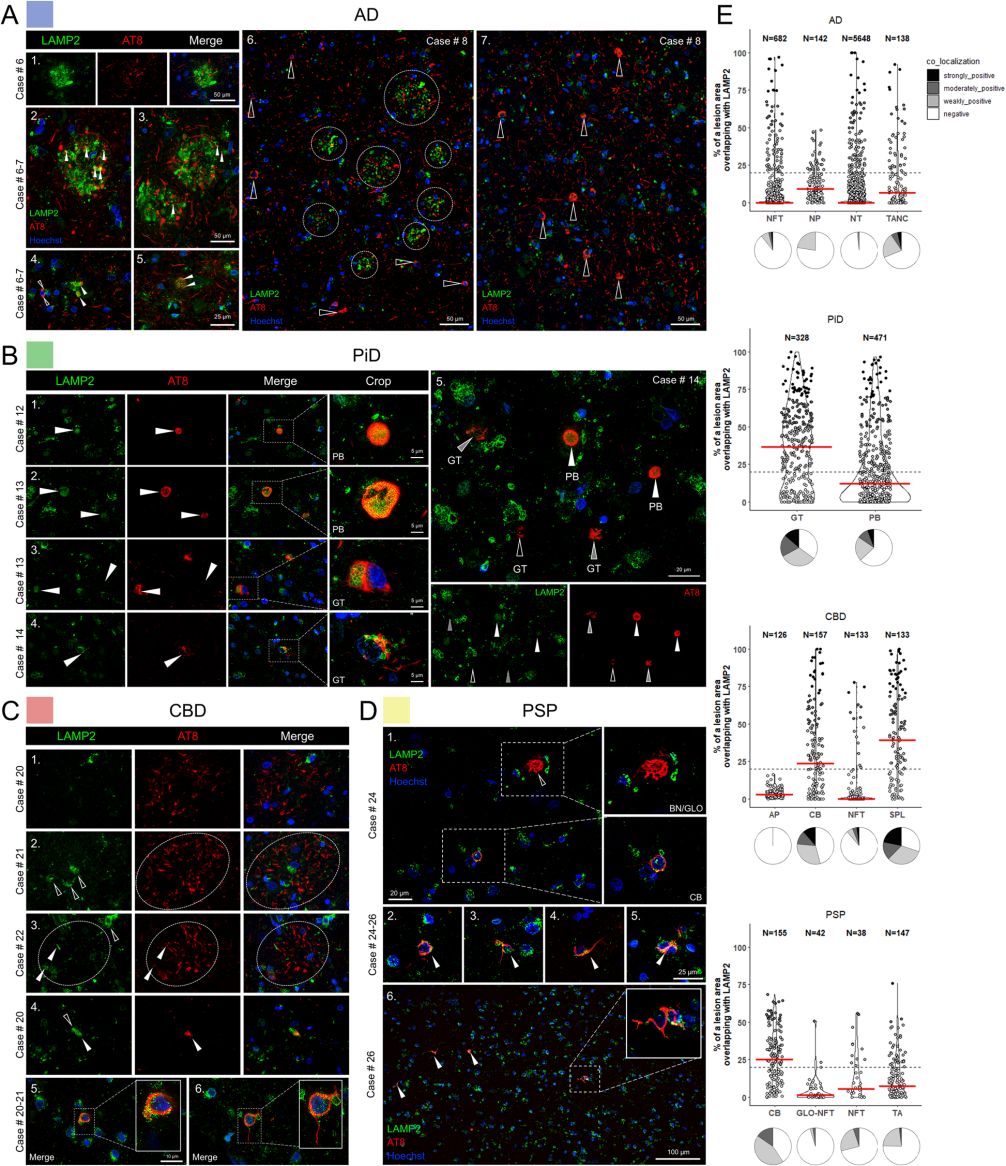
LAMP2 colocalizes with neuritic AD inclusions, Pick bodies and oligodendroglial coiled bodies in CBD and PSP frontal cortex. **A** In AD grey matter tissue, LAMP2 fluorescence delineated assemblies of engorged lysosomes, displaying spherical clusters of variably enlarged LAMP2-positive vesicles partially co-localized with phospho-tau structures (series 1–3, white arrowheads indicate co-localization). This abnormal and disease-specific distribution of LAMP2 protein in AD was reminiscent of AD NP pathology and was in proximity to AT8-positive NPs (series 2–3). Small TANCs and threads were also associated with LAMP2 (series 4–5). The robustness of both the distribution of LAMP2 clusters and to neuritic AD plaques can be observed in wide-view tile scans (series 6, white arrowheads) whereas NFT and NT pathology were mostly negative for LAMP2 signal (series 6, unfilled arrowheads). **B** PiD neuropathology showed LAMP2 accrual in the cores of grey matter PBs (series 1–2) and GTs found in the white matter (series 3–4). On the grey/white matter boundary LAMP2 was found within some GT lesions but not all (series 5, unfilled arrowheads—not colocalized, gray arrowheads –partially co-localized). **C** APs in CBD cases did not markedly sequester LAMP2 (series 1–3). Yet with larger and more mature APs, an increased presence of AT8 negative and LAMP2 laden cells was noted in addition to some small points of co-localization (series 2–3, open and white arrowheads, respectively). Small AT8 phospho-tau structures in CBD cases (SPLs) were associated with LAMP2 (series 4). Oligodendroglial inclusions were decorated with LAMP2 vesicles across CBD cases (series 5). **D** Neuronal PSP tau pathology (GLO NFTs) predominantly did not co-localize with LAMP2 (1, unfilled arrowheads). Some PSP TAs (series 3, 6) and CBs (series 1–2, 4–5) were weakly positive for LAMP2 signal across PSP cases. **E** Quantification of LAMP2-positive phospho-tau lesions from *n* = 3 cases/tauopathy showing the relative area of overlap between AT8 and LAMP2 in tauopathy lesions. Modified violin plots with data points show individual lesions plotted against relative overlap area. Lesions are categorized into co-localization categories based on their relative area of overlap. Red lines indicate median values. Pie charts underneath the violin plots illustrate the relative proportions of lesions with different degrees of co-localization. Abbreviations: LAMP2, lysosome-associated membrane glycoprotein 2; AD, Alzheimer’s disease; PiD, Pick’s disease; CBD, corticobasal degeneration; PSP, progressive supranuclear palsy; NFTs, neurofibrillary tangles; TANCs, tangle-associated neuritic clusters; APs, astrocytic plaques; CBs, coiled bodies; PBs, Pick bodies; GT, glial tau; NTs, neuropil threads; NP, neuritic plaque; SPL, small phospho-tau lesions; TA, tufted astrocytes; GLO-NFT, globose NFTs

LAMP2 is a protein of the lysosomal membrane that has a major role in lysosomal biogenesis, autophagic vacuole formation, phagosome maturation, endolysosome cholesterol trafficking and as a receptor for chaperone mediated autophagy (CMA) [114–116]. LAMP2 was selected for further examination across human tauopathies because the association of tau with LAMP2 has not been reported in previous studies of tau interactomes. Dense accumulation of LAMP2 swollen vesicles into clusters characterized the disease-associated nature and distribution of LAMP2 specifically in AD. LAMP2 clusters formed large concentric arrangements around AT8-positive neurites, some associated with NPs (Fig. 5A, E). While the majority of tangle associated neuritic clusters (TANCs) were partially or fully associated with LAMP2, it was not remarkably sequestered into NT of AD (Fig. 5A, E). To confirm that the LAMP2 clusters in AD were neuritic plaques, thioflavin-S co-staining highlighted neuritic plaques with both amyloid cores and AT8-positive neurites associated with LAMP2 (Supplementary Fig. 5A). Most NFTs in both AD and CBD cases were negative for LAMP2, with ~ 10% of them demonstrating some levels of overlap between AT8 and LAMP2 signals (Fig. 5E).

In PiD, over 30% of PBs and 60% of GTs, along with a very little percentage of NTs demonstrated co-localization with LAMP2 (Fig. 5B, E). CBD cases did not show significant association of LAMP2 with astrocytic plaques (Fig. 5C, E), while solitary cells in the perimeter of APs were positive for LAMP2, but not phospho-tau (Fig. 5C). In all the studied CBD cases there was association of LAMP2 with small phospho-tau lesions (Fig. 5C, 5E), and the majority of CBs had full or partial co-staining with AT8 and LAMP2 (Fig. 5C, E). PSP cases had association of LAMP2 with glial tau pathology in CBs and TAs from all cases. Globose NFTs were predominantly negative for LAMP2 (Fig. 5D, E), while minor portion of non-globose NFTs was weakly or moderately positive (Fig. 5E). As expected, normal control cases were negative for AT8 staining, but there was cytosolic vesicular localization of LAMP2 (Supplementary Fig. 5B). Neuropathologic characterization of VPS35 and LAMP2 corroborates the mass spectrometry proteomics findings, confirming their association with AT8-positive aggregates in all four major tauopathies. Nonetheless, we also demonstrate selective VPS35 and LAMP2 sequestration and abnormal accumulation into disease-specific phospho-tau lesions.

### ProPPr identifies differential association of proteins with tauopathy-specific pathology

**Fig. 6:**
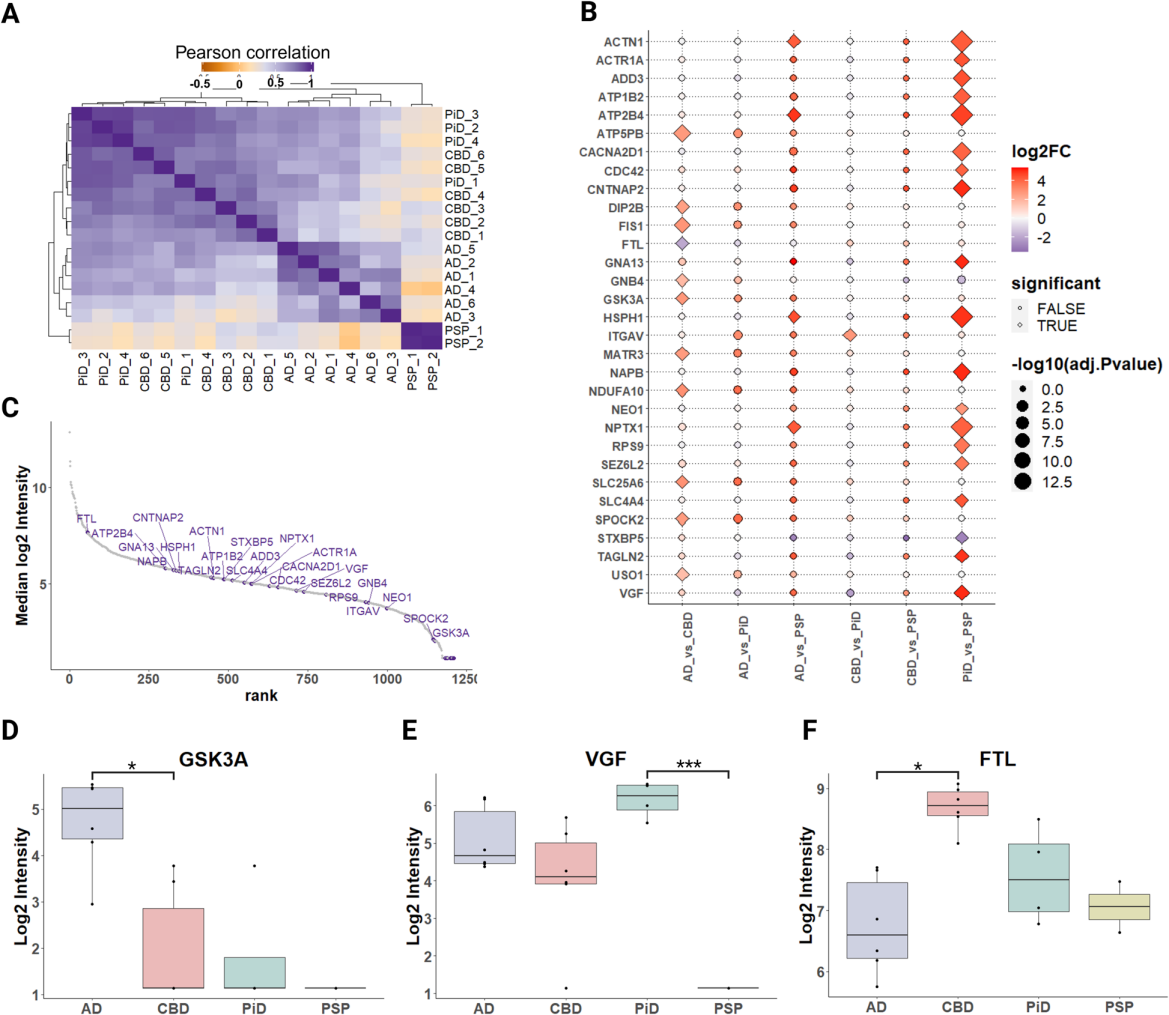
Analysis of differential protein association with phospho-tau lesions across tauopathies. **A** Sample correlation plot generated using abundance values for the proteins significantly changed in at least one of the pairwise comparisons between the tauopathies. **B** Log2 fold changes for proteins with significant changes in association with phospho-tau in each pairwise comparison presented as a dot plot. Diamond shape indicates statistically significant difference (adjusted *p*-value < 0.05). **C** Dynamic range of median protein intensities in the mass spectrometry proteomics data. Significantly changed proteins are shown in purple. **D** Summarized GSK3α protein intensity in tauopathies measured by mass spectrometry. **E** Summarized VGF protein intensity in tauopathies measured by mass spectrometry. **F** Summarized FTL protein intensity in tauopathies measured by mass spectrometry. Abbreviations: GSK3α, glycogen synthase kinase 3 alpha; FTL, ferritin light chain; AD, Alzheimer’s disease; PiD, Pick’s disease; CBD, corticobasal degeneration; PSP, progressive supranuclear palsy

To discover proteins with differential association with phospho-tau lesions between AD, CBD, PiD and PSP, we performed statistical comparison of protein abundances in samples from corresponding cases using the R package DEP. Since the MiST algorithm provides a “yes/no” type of answer to whether particular proteins interact with a studied bait based on the cut-off applied, it is not designed for quantitative comparisons of protein–protein interactions between several conditions. We identified 31 proteins with significantly different abundance (adjusted *p-*value <  = 0.05) in at least 1 of 6 pairwise comparisons cases and controls. Clustering analysis based on Pearson correlation revealed that the randomized samples from cases with the same pathological diagnosis generally clustered together, indicating that many of the identified significantly changed proteins could be characteristic features that distinguish different types of tau pathologies (Fig. 6A). These proteins were distributed along the range of protein abundances inferred from mass spectrometry data, suggesting that our analysis was not biased towards high or low abundance proteins (Fig. 6C). The largest differences were detected between PiD and. PSP (19 differentially abundant proteins), followed by differences between AD vs. CBD (11 proteins), AD vs. PSP (4 proteins) and CBD vs. PiD (1 protein). Comparisons between AD and PiD, as well as CBD and PSP, did not reveal any statistically significant protein differences in our dataset (Fig. 6B). These findings suggest that discernible differences in phospho-tau-associated proteomes between tauopathies can be detected by ProPPr. Careful neuropathologic analysis of disease-specific tau lesion is warranted to identify the association of identified proteins with specific tau aggregates. No significantly overrepresented pathways or GO terms were identified among the set of proteins with differential abundance between tauopathies. Consequently, we focused on a subset of individual proteins previously linked to neurodegenerative diseases for further analysis.

### GSK3α is associated with neuronal phospho-tau lesions

**Fig. 7:**
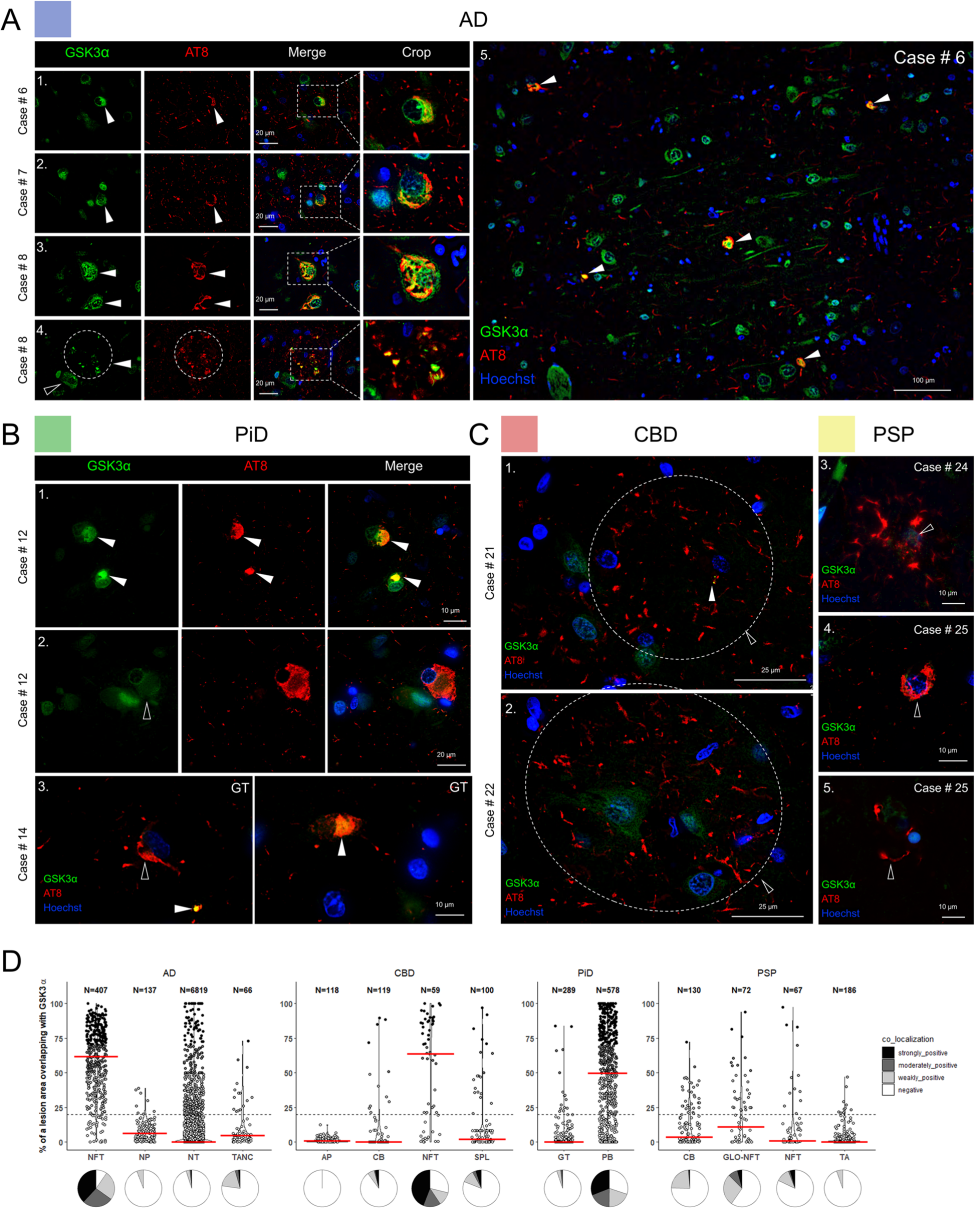
GSK3α is sequestered in distinct neuronal phospho-tau inclusions in tauopathy cortical tissue. **A** NFTs (series 1–3) and some phospho-tau components of NPs (series 4) co-localized with GSK3α in frontal cortex AD tissue tile image demonstrates the abundance of GSK3α and AT8 positive NFTs in AD [5]. **B** Some PiD phospho-tau inclusions were found to be positive for GSK3α, including neuronal PBs (series 1, white arrowheads) but not PC/BNs (series 2, unfilled arrowheads). White matter GT inclusions were either positive (white arrowheads) or negative (unfilled arrowheads) for GSK3α (series 3–4). **C** Astrocytic tau lesions in CBD and PSP mostly did not co-localize with GSK3α. **D** Quantification of GSK3α -positive phospho-tau lesions from *n* = 3 cases/tauopathy showing the relative area of overlap between AT8 and GSK3α in tauopathy lesions. Modified violin plots with data points show individual lesions plotted against relative overlap area. Lesions are categorized into co-localization categories based on their relative area of overlap. Red lines indicate median values. Pie charts underneath the violin plots illustrate the relative proportions of lesions with different degrees of co-localization. Abbreviations: GSK3α, glycogen synthase kinase 3 alpha; AD, Alzheimer’s disease; PiD, Pick’s disease; CBD, corticobasal degeneration; PSP, progressive supranuclear palsy; NFTs, neurofibrillary tangles; TANCs, tangle-associated neuritic clusters; APs, astrocytic plaques; CBs, coiled bodies; PBs, Pick bodies; Pick cells/BNs, Pick disease specific ballooned neurons; GT, glial tau; NTs, neuropil threads; NP, neuritic plaque; SPL, small phospho-tau lesions; TA, tufted astrocytes; GLO-NFT, globose NFTs

GSK3α was found to be increased in AD compared to CBD in our ProPPr data sets (Fig. 6B, F). GSK3α has been linked to AD pathology due to its ability to phosphorylate tau protein and to facilitate production of amyloid-beta-40 and amyloid-beta-42 peptides [117, 118]. To determine the localization of GSK3α in phospho-tau lesions, we co-immunostained post-mortem frontal cortices from patients with different tauopathies with anti-GSK3α and AT8. We found that GSK3α frequently co-localizes with AT8 in NFTs but rarely with AT8-positive NPs (Fig. 7A, E), while its distribution in cells without tau pathology was similar to control cases (Supplementary Fig. 6C). Neuronal PTs and mature tangles were both positive for GSK3α and AT8, while much less visible colocalization was observed in NTs (Fig. 7E, Supplementary Fig. 6A). We also found a co-localization of GSK3α with PBs in both grey and white matter of PiD (Fig. 7B, E Supplementary Fig. 6B). Examination of GT lesions in CBD and PSP frontal cortices revealed predominant absence of co-localization of GSK3α and AT8-positive phospho-tau (Fig. 7C-E,). Association with GSK3α was still observed in NFT in CBD and less frequently in PSP. Our results demonstrate that GSK3α is sequestered predominantly in neuronal inclusions across tauopathies, while it is mostly absent from glial lesions in PSP and CBD. The increased abundance of GSK3α in AD in proteomics data is most likely explained by the higher proportion of neuronal phospho-tau pathology in AD compared to CBD and PSP, and higher tau burden compared to PiD.

### VGF is associated with Pick bodies and Alzheimer’s disease neurofibrillary pathology

**Fig. 8:**
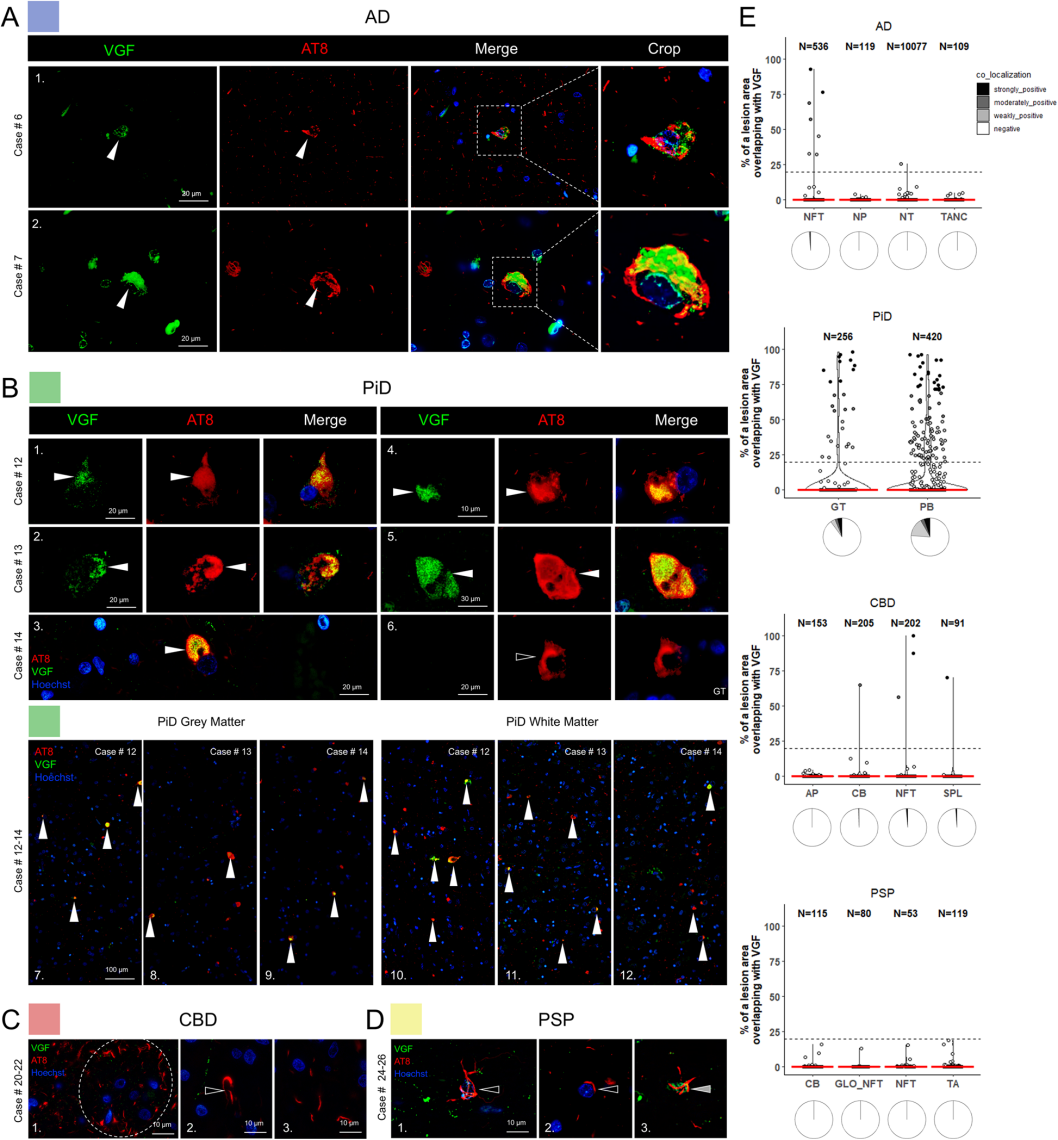
VGF co-localizes with AT8 phospho-tau aggregates in AD and PiD human autopsy cases. **A** In AD cases, VGF was occasionally sequestered into NFTs (series 1–2). **B** Neuronal PiD inclusions were co-localized with VGF, which associated within the cores of most PBs, ranging from spherical to horseshoe-shaped (series 1–5). Glial inclusions were mostly found not to be associated with VGF (series 6). 60 × tile imaging demonstrates the association of sequestered VGF to grey matter PBs (series 7–9) and variable white matter glial inclusions (series 10–12). **C** VGF did not co-localize in APs of CBD (series 1), and most oligodendroglial CBs (series 2), or NT pathology (series 3). **D** PSP TAs (series 1) and CBs (series 2) did not co-localize with VGF. Very rare instances of partial co-localization below the chosen 20% cut-off overlap were observed in a minority of TAs in one PSP case (series 3). **E** Quantification of VGF -positive phospho-tau lesions from *n* = 3 cases/tauopathy showing the relative area of overlap between AT8 and VGF in tauopathy lesions. Modified violin plots with data points show individual lesions plotted against relative overlap area. Lesions are categorized into co-localization categories based on their relative area of overlap. Red lines indicate median values. Pie charts underneath the violin plots illustrate the relative proportions of lesions with different degrees of co-localization. Abbreviations: AD, Alzheimer’s disease; PiD, Pick’s disease; CBD, corticobasal degeneration; PSP, progressive supranuclear palsy; NFTs, neurofibrillary tangles; TANCs, tangle-associated neuritic clusters; APs, astrocytic plaques; CBs, coiled bodies; PBs, Pick bodies; GT, glial tau; NTs, neuropil threads; NP, neuritic plaque; SPL, small phospho-tau lesions; TA, tufted astrocytes; GLO-NFT, globose NFTs

The neurosecretory protein VGF (non-acronymic) has emerged in large-scale -omics studies as a potential biomarker of several neurodegenerative and psychiatric diseases [119]. In our ProPPr data, VGF had a significantly higher association with phospho-tau lesions in PiD compared to PSP with a trend for increase compared to other tauopathies (Fig. 6B, E). To further examine this finding, we performed co-immunofluorescence of VGF with phospho-tau in the four major tauopathies, as well as normal controls (Fig. 8 and Supplementary Fig. 7). In AD cases, VGF was rarely present in NFTs, including both PTs and mature NFTs (Fig. 8A, E). There was no association of AD NTs with VGF, and we did not find a co-localization between VGF and neuronal or glial lesions in PSP (Fig. 8D). In CBD the vast majority of phospho-tau inclusions were VGF-negative (Fig. 8C), although very rare associations with VGF were observed in CBs, NFTs and small p-tau lesions (Fig. 8E). In contrast, we found a stronger accumulation of VGF in a portion of 3R-tau PBs found in PiD frontal cortex, where VGF was located in the core of PBs and sometimes surrounded by AT8-positive tau (Fig. 8B, E). Sparse glial inclusions in the white matter also showed co-localization of VGF and tau (Fig. 8B, E). The frequency of VGF co-localization with PiD PBs was significantly higher compared to all other lesions examined (Supplementary Fig. 8). Thus, a predominant VGF association with pathologic tau in PiD confirms observations made with ProPPr. In controls, there was punctate neuropil labeling with VGF and occasional neurons with strong perikaryal VGF staining (Supplementary Fig. 7). Of note, young control tissue (Table 1. Case #35) had higher levels of VGF in grey matter than aged cases (Supplementary Fig. 7).

### FTL-positive microglia are associated with phospho-tau in CBD astrocytic plaques

**Fig. 9:**
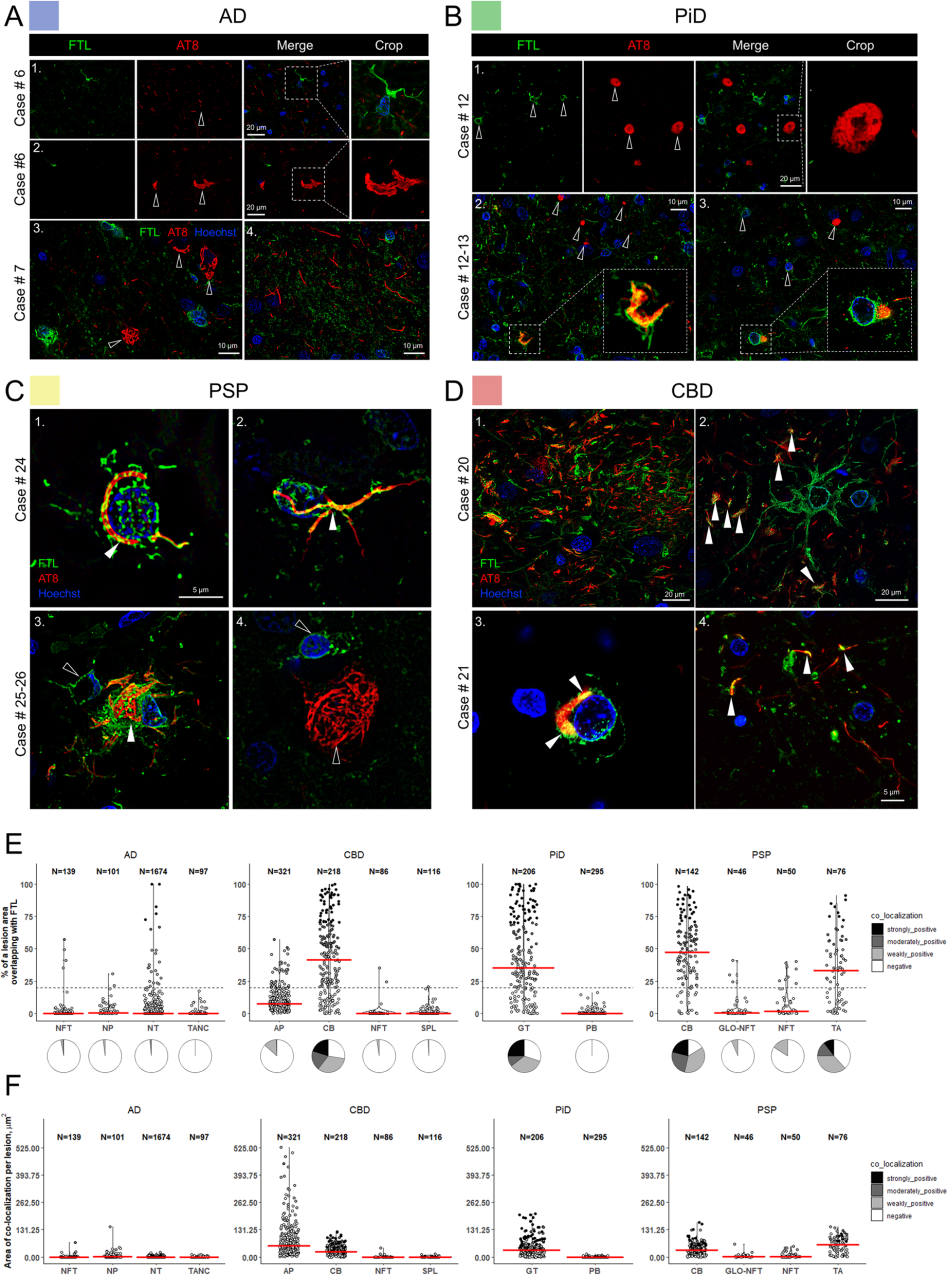
FTL co-localizes with glial tau pathology in cortical tissue from primary tauopathy cases. **A** FTL was highly enriched in cells with a microglia-like morphology in AD tissue (series 1) and did not co-localize NFTs (series 2–3) or other AD neuritic pathology (series 4). **B** PiD neuronal inclusions were negative for FTL (series 1–3, unfilled arrowheads). Glial inclusions located in the white matter were markedly co-localized with FTL (series 2–3). **C** PSP CBs (series 1) and some TAs (series 2–3) were positive for FTL and AT8, while GLO-NFTs did not associate with FTL signal (series 4). **D** CBD APs showed strong co-localization within the corona of tau positive distal extensions with FTL decorating various sizes of APs (series 1). In most cases, FTL-positive cells were often found close to the center of developing APs (series 2). FTL also co-localized with CBs (series 3), and some NTs (series 4). **E** Quantification of FTL-positive phospho-tau lesions from *n* = 3 cases/tauopathy showing the relative area of overlap between AT8 and FTL in tauopathy lesions. Modified violin plots with data points show individual lesions plotted against relative overlap area. Lesions are categorized into co-localization categories based on their relative area of overlap. Red lines indicate median values. Pie charts underneath the violin plots illustrate the relative proportions of lesions with different degrees of co-localization. **F** Quantification of absolute areas of overlap between AT8 and FTL per individual tauopathy lesions from *n* = 3 cases/tauopathy, Modified violin plot with data points show individual lesions plotted against absolute overlap area. Lesions are categorized into co-localization categories as in E. Red lines indicate median values. Abbreviations: AD, Alzheimer’s disease; PiD, Pick’s disease; CBD, corticobasal degeneration; PSP, progressive supranuclear palsy; NFTs, neurofibrillary tangles; TANCs, tangle-associated neuritic clusters; APs, astrocytic plaques; CBs, coiled bodies; PBs, Pick bodies; GT, glial tau; NTs, neuropil threads; NP, neuritic plaque; SPL, small phospho-tau lesions; TA, tufted astrocytes; GLO-NFT, globose NFTs

The proteomics results also point to a strong association of ferritin light chain (FTL) with phospho-tau in CBD (Fig. 6B, D). The ferritin complex functions to keep iron in a soluble and non-toxic form. Mutations in the *FTL* gene lead to neuroferritinopathy, an autosomal dominant neurodegenerative disease with significant brain iron accumulation [120–122]. In AD cases, AT8-positive pathology including NFT, PT, NP, TANC and NT were predominantly negative for FTL by immunofluorescence imaging, although there were FTL-positive small cells resembling microglia present in the vicinity of NFTs in AD (Fig. 9A, E). This observation aligns with the known disease-associated microglial involvement of AD [123] and the previous observations that glial cells were positive for FTL (Fig. 9B-D). In PiD, FTL was not detected in PBs, but in GTs in the white matter (Fig. 9B, E), most likely representing 4R-tau positive astroglial inclusions [6, 124, 125]. FTL showed frequent co-localization with PSP CBs but was almost never present in GLO-NFTs or regular NFTs (Fig. 9C). Similar to PSP, CBs in CBD cases demonstrated the highest frequency of high degree co-localization with FTL (Fig. 9D, E). In addition, we observed frequent and robust co-localization of FTL with CBD APs (Fig. 9D, E). Although most of the APs fell into the categories of “negative” and “weakly positive” based on relative overlap area measurements and our selected criteria, measurement of an absolute overlap area demonstrated that most of the co-localization between AT8 phospho-tau and FTL in CBD frontal cortices occurs within the astrocytic plaques (Fig. 9F).

The higher level of FTL association with phospho-tau lesions detected by proteomics in CBD compared to AD and to a lesser extent other tauopathies, is likely driven by the high abundance of FTL + APs in CBD.

**Fig. 10:**
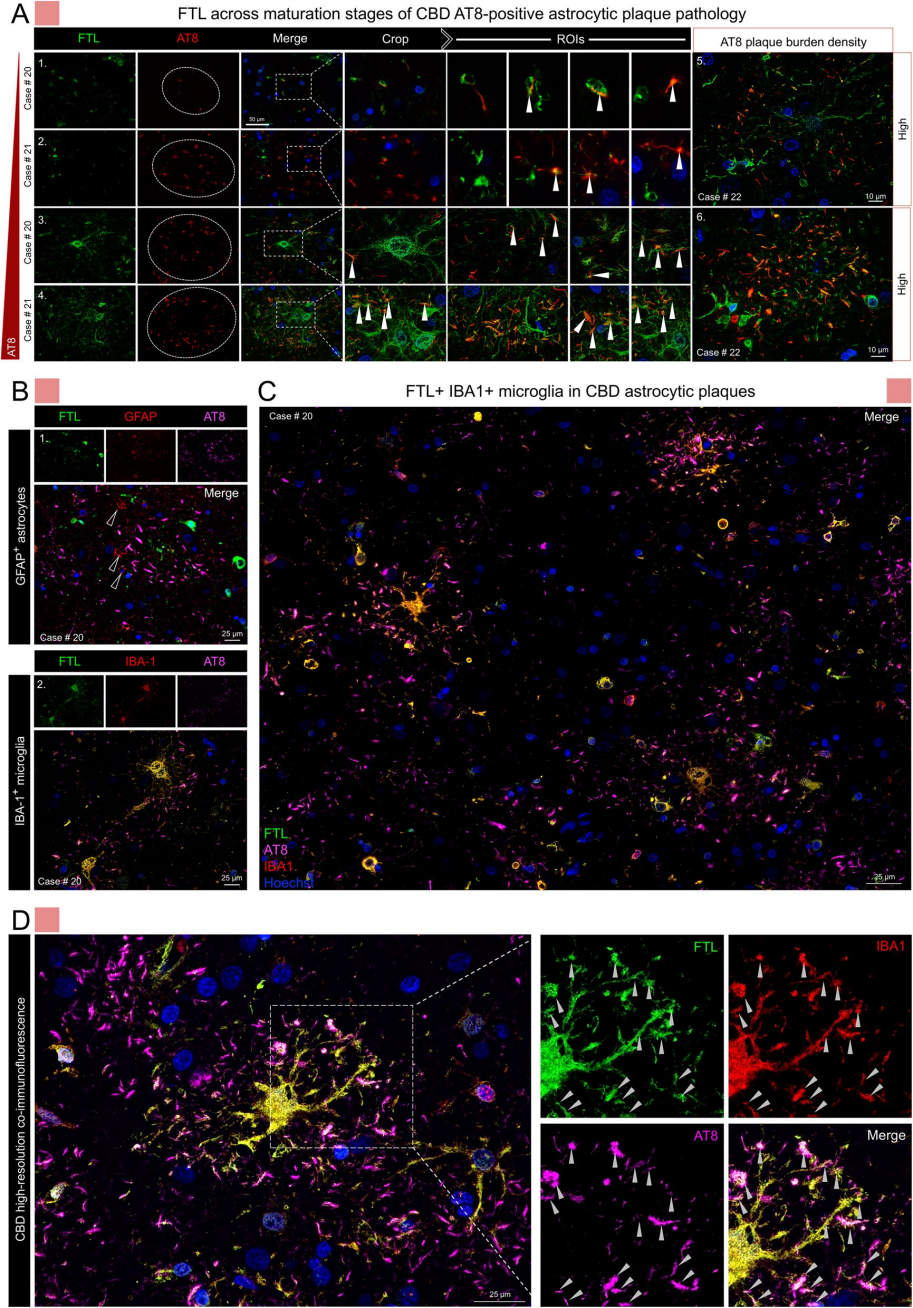
Astrocytic plaques in CBD frontal cortex associate with FTL laden microglia. **A** FTL-positive cellular structures are associated with AT8 phospho-tau in small (series 1–2, 5) and large (series 3–4, 6) APs. In larger and more mature APs FTL-positive cells are increased in size and become localized in proximity to a center of APs (series 3–4). **B** Triple labelling of FTL (green), AT8 (magenta), and GFAP (red, series 1) or IBA1 (red, series 2) show that large FTL-positive cells located in the centers of mature APs have microglial, but not astrocytic identity. **C** Tile image of triple FTL/AT8/IBA1 labeling across CBD cortical tissue showing the frequency and extent of co-localization between FTL laden microglia and phospho-tau APs. **D** High magnification image of an FTL + microglia cell infiltrating an astrocytic plaque. Grey filled arrowheads denote processes with FTL/AT8/IBA1 triple co-localization. Abbreviations: CBD, corticobasal degeneration; FTL, ferritin light chain; IBA1, microglial marker ionized calcium-binding adapter molecule 1; GFAP, glial fibrillary acidic protein; APs, astrocytic plaques

In addition, while examining CBD frontal cortex sections we have often observed large FTL-expressing cells were centrally located in APs (Fig. 9D). Upon examination of astrocytic plaques of different sizes, likely reflecting tau accumulation during AP maturation, smaller APs had nearby FTL-positive cells instead of a robust co-localization with FTL in mature AP (Fig. 10A). This observation suggested that instead of FTL upregulation in astrocytes affected by tau aggregation, the primary source of FTL may be from other cells that respond to tau pathology, such as microglia. To test this hypothesis, we performed triple co-immunofluorescent labeling of CBD frontal cortices with AT8, anti-FTL antibodies, and antibodies to either astrocyte (GFAP) or microglia (IBA1) cell-type specific markers (Fig. 10B-C). We found that AT8 co-localized with FTL in large and mature plaques, which were negative for GFAP, but positive for IBA1 (Fig. 10B). IBA1 + and FTL + positive microglia demonstrated a strong co-localization and an extensive level of dystrophic cellular morphology. These APs revealed fragmented GFAP positive extensions, lacking an observable cell soma, and instead showed IBA1 + /FTL + cells in the center of AP (Fig. 10B). AT-8 and IBA1 positive processes suggest a potential engulfment of phospho-tau by microglia and its uptake via phagocytosis (Fig. 10B). High-magnification tiled imaging of multiple FOVs showed that FTL-positive microglial cells were often located within the central core of APs, but also between APs in the neuropil and closely associated to regions of phospho-tau pathology. APs may recruit microglia and induce an FTL-positive dystrophic microglial phenotype upon AP colonization during the maturation of AP pathology. Taken together, ProPPr identifies discernible differences in phospho-tau-associated proteomes between tauopathies that reflect distinct molecular mechanisms underlying pathological tau aggregation in different disease conditions.

## Discussion

Here we describe the development and first application of our ProPPr method that has been optimized for in situ proximity proteomics on FFPE tissue sections mounted on glass slides. Using this method, we have performed the first comprehensive ProPPr profiling of the phospho-tau-associated proteome across four major tauopathies – AD, CBD, PiD, and PSP. While this strategy is related to various assays described before [65, 66, 68, 69], it has several important modifications. Firstly, instead of frozen or fixed 50–85 μm thick vibratome sections, for each studied case we used a small number of 5 μm FFPE tissue sections mounted on standard glass slides, which are readily available from brain banks, allow for automated workflows with autostainers, and are expected to increase the level of antibody penetration and protein labeling density. Secondly, for tissue lysis and reversal of crosslinking we used buffers based on the MS-compatible surfactant RapiGest instead of commonly used SDS-containing buffers [126]. Finally, the utilization of biotin-SS-tyramide that contains a disulfide bond that is cleavable by reducing agents allowed us to elute purified biotinylated proteins from streptavidin beads. Since biotinylated proteins are normally lost due to the extraordinarily high affinity of streptavidin for biotin tags, this strategy potentially increases the yield and specificity of the affinity purification method. In addition, we selected five proteins identified through proteomics for an extensive investigation of their associations with heterogenous phospho-tau lesions. Fluorescence IHC combined with quantitative co-localization analysis at the level of individual lesions provided valuable insights into the differential association of the studied proteins with various types of tau pathologies. In proximity proteomics experiments, the total quantity of a bait-associated protein detected by mass spectrometry directly reflects the number of protein molecules located in close proximity to the bait. In the context of heterogeneous tau pathology in post-mortem human tissue, this value is influenced by several factors, including the frequency of distinct lesions, their cell-type specificity, and the area they occupy. The observed complex co-localization patterns underscore the importance of follow-up IHC validation of proximity proteomics findings in complex human pathologies such as tauopathies.

We report the identification of 1,317 phospho-tau-associated proteins from 1,314 genes, 409 of which were not reported in previous mass spectrometry proteomic studies investigating phospho-tau interactors and NFT components [58, 59, 69]. We found 229 proteins to be associated with phospho-tau in all four major tauopathies: AD, CBD, PiD, and PSP. One of the largest groups of interrelated GO terms overrepresented in this common interactor list is associated with protein folding. These categories include proteins that belong to several groups of molecular chaperones: HSP70, HSP90, CCT/TriC, and chaperone-like proteins CLU and VCP. The proteins from these groups, including the ones identified by us, were previously found in tau interactome and NFT proteome studies [58, 59]. Moreover, CLU has been previously shown to co-localize with tau deposits in AD, CBD, and PiD post-mortem brains, while HSP70 and HSP90 chaperones were found to be sequestered into seeding-induced tau aggregates in vitro [127, 128]. Recruitment of molecular chaperones into phospho-tau aggregates likely reflects the response of cellular folding machinery to the emergence of misfolded tau species. It has been shown that different chaperones can effectively reduce tau phosphorylation, aggregation, and filament formation [127, 129–132]. On the other hand, it has also been demonstrated that chaperone-activity can stabilize amyloidogenic tau folding, facilitate tau filament formation or increase production of seeding-competent tau forms, thus promoting the spread of tau pathology [133–139]. Therefore, association of molecular chaperones with tau lesion may have opposite effects on the progression of tau pathology. In any case, sequestration of molecular chaperones in tau aggregates likely aggravates the proteostasis imbalance of aging proteomes.

For further studies we selected candidate proteins that were either common phospho-tau interactors or showed differential association with tauopathy-specific pathology. Co-immunofluorescence of postmortem frontal cortices confirmed their association with AT8-positive aggregates across the major tauopathies, demonstrating selective sequestration into disease and cell type specific phospho-tau lesions. The analysis of phospho-tau associated proteomes revealed major sequestration of lysosomal proteins in tau aggregates in all the studied tauopathies, including multiple V-type proton ATPase subunits and the LAMP2. Interestingly, recent study demonstrated the ability of phosphorylated tau to disrupt V-type proton ATPase function through its interaction with the ATP6V1B2 subunit [140], which was associated with phospho-tau lesions in all 4 studied tauopathies. Of note, we also found that LAMP2 co-localizes with AT8 phospho-tau in a variety of phospho-tau lesions across tauopathies, most notably in AD TANCs, CBD and PSP CBs, and both glial and neuronal aggregates in PiD. The sequestration of LAMP2 in PBs has also been reported in an earlier study [141]. Lysosomes play an important role in the clearance of tau aggregates as part of the autophagy-lysosome system, suggesting that the observed association of tau aggregates with lysosomal proteins may reflect the failure of cellular mechanisms to clear tau aggregates at advanced disease stages [142]. The sequestration of lysosomal proteins in tau aggregates may lead to lysosomal dysfunction, either as a result of protein displacement from lysosomal membranes or the entrapment of intact lysosomes in phospho-tau aggregates. Treatment of cultured human astrocytes with preformed tau fibrils results in lysosome deacidification and swelling, suggesting that pathological tau aggregates can adversely affect lysosomal activity and, more broadly, the endolysosomal system [143]. Our findings together with earlier studies identifying endolysosomal system defects in brains from AD and other tauopathies and phenotypes observed in in vitro tauopathy models, suggest a key role of this pathway in human tauopathies [110, 144].

Another protein that was found associated with tau in all four major tauopathies is the core retromer complex protein VPS35. While this protein has been previously found in the proteome of NFTs in AD and identified as a tau interactor in several studies, its specific association with pathological lesions in primary tauopathies has not been reported [59, 105, 108, 145, 146]. We found an abnormal accumulation of VPS35 in several types of phospho-tau lesions in frontal cortices from all the studied tauopathies. It appears likely that the sequestration of VPS35 into insoluble tau aggregates may lead to retromer complex deficiency. In support of this, several lines of evidence suggest the impairment of retromer functioning in tauopathies. Retromer dysfunction in AD is indicated by reduction of VPS35 and VPS26 protein levels in the entorhinal cortex that is particularly vulnerable for tau pathology in AD [147]. A similar phenomenon was also observed in brains of young and older Down syndrome patients who have increased risk of developing AD neuropathology after the age of 40 [148]. In addition, the known retromer complex receptor SORL1 is decreased in trans-entorhinal cortex in AD, which may be caused by an insufficiency of SORL1 recycling by the retromer complex [149]. Finally, the retromer complex is known to play an important role in regulating lysosomal homeostasis [150], and lysosomal defects are well documented in both AD and primary tauopathies, as discussed above [110, 144]. Altogether, these data suggest that retromer complex disfunction is a characteristic feature of tauopathies, and VPS35 sequestration into phospho-tau pathological lesions may play an important role in pathogenesis of these disorders. Importantly, evidence shows the detrimental effect of retromer dysfunction and beneficial effect of restoration of the retromer complex function on tau pathology [151–156]. This may suggest the existence of a positive feedback loop between tau aggregation and retromer dysfunction. Another retromer subunit, VPS29, was found to be a strong modifier of tau propagation in iPSC-derived glutamatergic neurons, suggesting that retromer complex dysfunction may also facilitate spreading of tau pathology [157].

Aside from the common phospho-tau associated proteins, we also selected candidates with differential association with phospho-tau for further analysis with immunofluorescence imaging. Using this approach, we found accumulation of GSK3α in NFTs in AD and in PiD PBs. Phosphorylation of tau by GSK3 kinases leads to its dissociation from microtubules, potentially contributing to pathological tau aggregation and neurodegeneration [118, 158, 159]. While GSK3α is one of the kinases that are known to phosphorylate tau at sites relevant to pathological tau aggregation, including S396, T231, S235 and S202, relatively little is known about its role in tau pathology compared to its isozyme GSK3β, indicating the need for further studies [118].

We also demonstrated sequestration of the neurosecretory protein VGF into some tau aggregates in AD (NFTs) and especially in PiD (PB and GT inclusions). Recently, VGF has been identified as interactor of wild-type and mutant forms of tau in an in vitro proximity proteomic study [108]. In physiological conditions, VGF is proteolytically processed in neuronal Golgi apparatus and secretory vesicles to produce active VGF-derived peptides that are then secreted by exocytosis to stimulate responses in neighboring cells [119]. Functions of these peptides include, but not limited to, synaptogenesis, formation of long-term memory in hippocampus, and activation of microglia [160–162]. While we cannot draw a definitive conclusion from our proteomic data on which VGF proteoforms are sequestered in phospho-tau lesions, it is most likely the VGF precursor since some of the identified peptides lie outside of the boundaries of known VGF-derived peptides. Antibodies used in the immunofluorescent analysis were raised against central part of the VGF precursor molecule that contain only a few known active peptides, also indicating that their most likely target is the VGF precursor molecule. Sequestration of the VGF precursor into tau lesions may result in disruption of active VGF-derived peptide production and secretion, potentially playing a crucial role in neurodegeneration. In support of this hypothesis, reduction of VGF peptides in CSF and brain has been reported in different neurodegenerative conditions, including AD and frontotemporal dementia (FTD) [163–174]. This defect may at least partially result from insufficient secretion of VGF-derived active peptides, since VGF-derived peptide TLQP-62 has been shown to stimulate VGF translation through VGF/BDNF/TrkB or mTOR/GPCR-dependent autoregulatory feedback loops [175, 176]. Of note, no change in VGF protein level was detected in PSP, while our study has found no association between VGF and PSP phospho-tau lesions, supporting a link between VGF sequestration into aggregates and VGF active peptide levels in disease [174].

VGF is increasingly recognized as a major player in neurodegenerative diseases. Most notably, VGF was recently identified as a key driver of AD by multiscale casual network analysis of multiomics data generated as part of the Accelerating Medicines Partnership-AD (AMP-AD) program from a large cohort of late-onset AD and control individuals [177]. The importance of VGF for AD pathology is supported by the findings that VGF partially mediates the effect of AD polygenic risk score on cognitive decline, with an effect size comparable only to beta-amyloid and tau [178]. A recent study established a link between higher *VGF* expression in neurons and slower cognitive decline and lower odds of neurodegenerative pathologies in older adults [179]. This makes VGF not only a promising biomarker for neurodegenerative diseases, but also a prospective target for therapy. It has been shown that increase of VGF levels by VGF overexpression or upregulation of VGF translation rescued or partially rescued number of phenotypes in 5xFAD mouse model of AD [177] and that administration of VGF-derived peptide TLQP-21 in the same mouse model produced similar results [180]. Our results, however, suggest that similar therapeutic approach can be extended from AD-associated amyloid plaque pathology to tauopathies, particularly PiD. Although there are currently no well-established animal models for 3R tauopathies, it would be interesting to test the effect of different modes of VGF administration in available tauopathy models, such as PS19 mouse line.

Our proteomics analysis also uncovered that FTL is more strongly associated with AT8 phospho-tau in CBD than in AD, PiD, or PSP. IHC analysis revealed that abundant FTL positive tau lesions in APs in CBD. We also found co-localization of phospho-tau with FTL in TAs in CBD and CBs in CBD and PSP. In agreement with our findings, FTL was recently identified as a phospho-tau interactor in brains from PSP [69]. We also found FTL co-localized with GTs in PiD. No co-localization was detected in neuronal lesions (NFTs, GLO-NFTs, PBs, NPs and NTs). This suggests that co-localization with FTL is a feature of glial tau pathology, in agreement with the evidence that FTL subunit is mostly expressed by glial cells, while neurons mostly express ferritin heavy chain subunit [181].

Our finding of higher FTL association with APs is in good agreement with the previous work that found higher level of FTL in CBD compared to PSP in the caudate nucleus [182]. This work also showed co-localization of FTL with APs and TAs. We now extend this finding to frontal cortex and show that association of FTL with phospho-tau is higher in CBD not only compared to PSP, but also to PiD and AD. In this study, however, the increase of FTL in the caudate nucleus in CBD was attributed to increase in FTL production by astrocytes [182]. Here we show that the majority of FTL within APs is associated with IBA1-positive microglia rather than within astrocytes. The difference between our results may arise from regional differences between frontal cortex and caudate nucleus, but also from the fact that different microglial markers have been used for co-immunostaining of microglia with FTL. While the previous study used HLA-DR as a microglial marker [182], HLA-DR and FTL-positive microglia have differences in morphology, suggesting that they may represent distinct microglial subpopulations [183]. In this study we used IBA1, which has been shown to be expressed in high level in microglia subpopulations with high expression of FTL [184].

Our study demonstrates that infiltration of APs with FTL-positive microglia is a characteristic feature of CBD pathology. High FTL expression in microglia has been previously suggested to be a marker of dystrophic microglial phenotype [183], which is in agreement with the morphological appearance of dystrophic microglia in CBD APs in this study. It is not entirely clear what exactly causes increased microglial FTL expression and dystrophic phenotype. One possibility is imbalance in iron metabolism, as it has been shown that FTL-positive microglia have higher iron load than other microglial subtypes. We did not evaluate iron load in our CBD cases in this study, but in a previous report, staining with Prussian blue did not detect differences in iron content that could explain changes in FTL expression between CBD and PSP [182]. It has also been hypothesized that dystrophic microglial phenotypes can be caused by microglia exhaustion associated with unsuccessful attempts to phagocyte insoluble protein aggregates [185]. This is supported by the presence of AT8-positive microglial processes in APs. Microglia can internalize tau fibrils directly, or via tau oligomer-containing synapses and neurons for phagocytosis [186–188]. The neurodegeneration-associated increase in FTL-positive microglia is likely to play an important role in disease progression [189], and it will be important to assess their role in CBD pathology in future studies.

In summary, this study introduces the ProPPr in situ proteomics method for profiling the composition and molecular environment of detergent-insoluble aggregates and applies it to the phospho-tau associated proteomes in distinct tauopathies. While there is abundant information on the complex morphologies, isoform composition, and fibrillar structure of tau in AD and primary tauopathies, there is a lack of insight into their proteomic composition. The ProPPr approach addresses the limitations of existing methods of local proteomic studies (co-immunoprecipitation, LCM, BioID) and allows for unbiased proteome-wide discovery in post-mortem tissue with the resolution of microscopy, making it applicable for studying human disease-associated protein pathology. The identified phospho-tau-associated proteomes complement the large existing body of data on tau interactomes in normal and pathological contexts obtained from a variety of disease models using different methods [59, 69, 103–108, 145, 146, 190–192]. This study also provides extensive neuropathologic evaluation of selected candidates of interest, showing intricate patterns of phospho-tau association with specific subtypes of lesions, with important implications for their specific roles in the disease process of tauopathies. Further studies and more advanced model systems will be needed to determine the functional role of identified proteins as potential disease modifiers, mediators of aggregate toxicity due to sequestration, and markers of disease-specific pathological lesions in tauopathies.

While our study represents the first attempt of comparative analysis of phospho-tau-associated proteome between tauopathies, it is subject to certain limitations that require consideration. A modest sample size in our experimental cohort likely led to an underestimation of the number of proteins exhibiting significantly different associations with phospho-tau aggregates among the tauopathies. Subsequent investigations involving larger cohorts may yield additional insights into both common and tauopathy-specific proteomes of pathologic tau lesions. Furthermore, the efficiency of the ProPPr method can be further improved by using ultra-sensitive mass spectrometry instruments for single-cell proteomics and refined techniques of protein extraction from FFPE tissue, such as focused ultrasonication [193]. Nevertheless, our study provides a comprehensive overview of phospho-tau-associated proteome in a range of tauopathies, and it uncovered several important findings that provide novel insight towards the pathological events and features that are common or specific to neurodegenerative tauopathies.

## Supplementary Information

Supplementary Material 1.

Supplementary Material 2.

Supplementary Material 3.

Supplementary Material 4.

Supplementary Material 5.

Supplementary Material 6.

## Data Availability

All data generated or analyzed during this study are included in this published article and its supplementary information. Raw mass spectrometry data is available on request and can be found at https://www.synapse.org/Synapse:syn64964787/files/.
