# Supplementary material for: Probe-dependent Proximity Profiling (ProPPr) Uncovers Similarities and Differences in Phospho-Tau-Associated Proteomes Between Tauopathies
Source: Mol Neurodegener. 2025 Mar 13;20:32. doi: 10.1186/s13024-025-00817-0 (PMC11905455; doi:10.1186/s13024-025-00817-0)
Supplement: Supplementary file 1 — Supplementary Material 1. [file 13024_2025_817_MOESM1_ESM.docx]

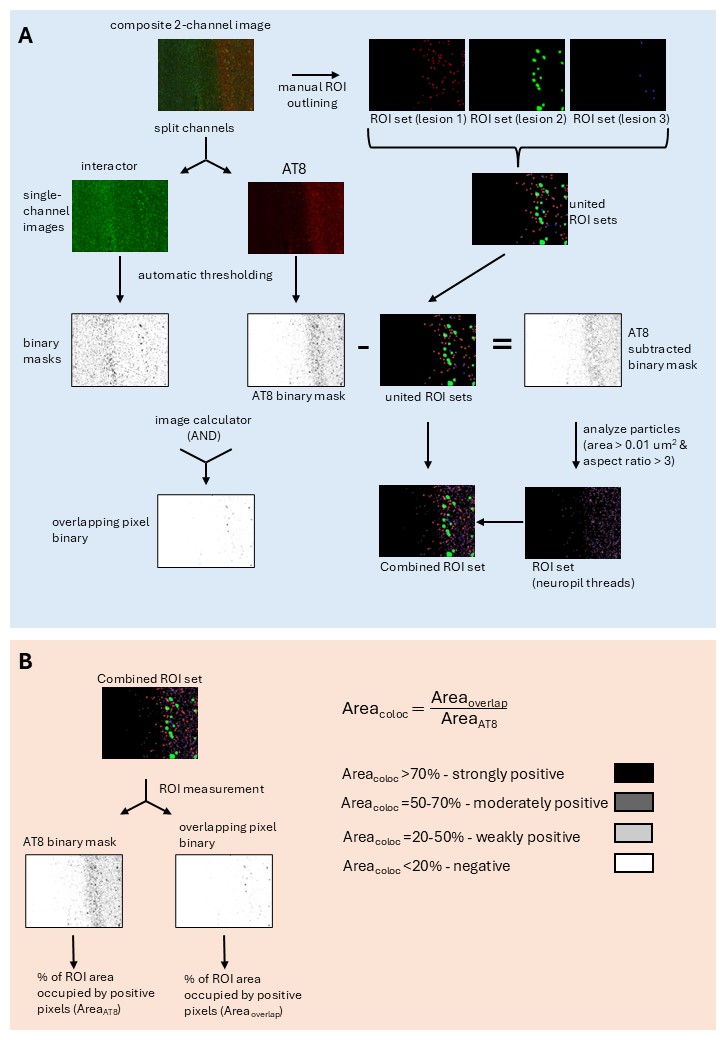


**Supplementary Fig. 1. Pipeline for co-localization analysis. (A)** Image pre-processing workflow. Tiled 5x5 field-of-view multichannel composite images were split into single-channel images and converted into binary images using automatic thresholding. Binary masks for AT8 and the phospho-tau-associated protein were combined to create a third binary mask containing overlapping pixels. Additionally, individual tau lesions (excluding NTs) were manually outlined by a neuropathology expert (MCW), generating sets of ROIs for each lesion type. For NT segmentation, ROI sets were combined into a binary mask and subtracted from the AT8 binary mask. Individual objects in the resulting binary image were segmented using Fiji’s “Analyze Particles” function, with thresholds set at an area > 0.01 μm² and an aspect ratio (longest to shortest dimension) > 3 to identify NTs. **(B)** Quantification of co-localization. Each ROI set was overlaid on AT8 and overlapping-pixel binary images. For each ROI, the percentage of its area occupied by positive pixels was calculated. Co-localization was classified as strongly positive (>70% overlap), moderately positive (50–70% overlap), weakly positive (20–50% overlap), or negative (<20% overlap), based on the ratio of the overlapping-pixel area to the total AT8-positive pixel area.


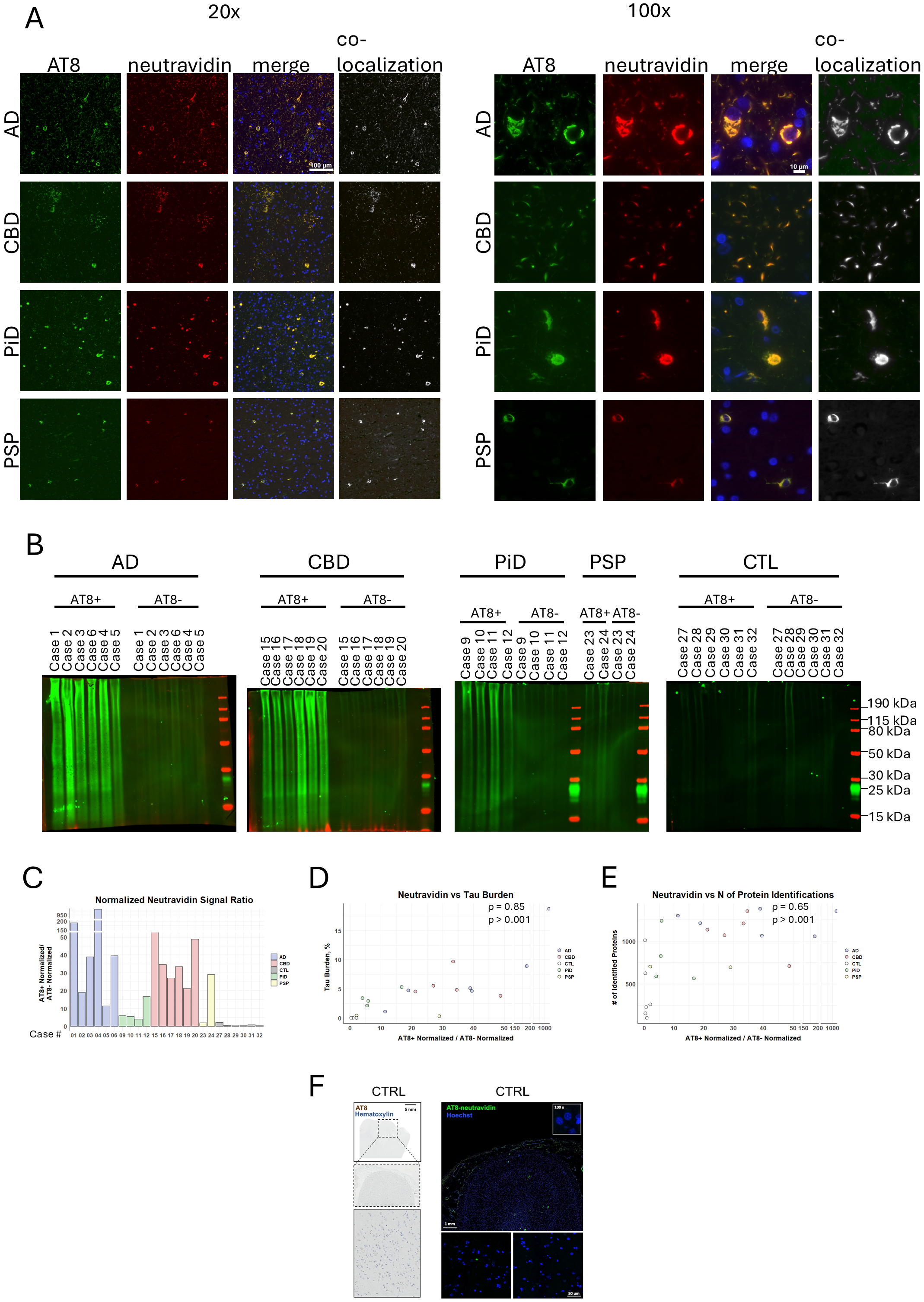


**Supplementary Fig. 2 | Specificity controls for the ProPPr method. (A)** Co-staining of frontal cortices from tauopathy cases that underwent AT8-mediated ProPPr proximity labelling with Alexa Fluor 488 goat anti-mouse antibodies (green) and neutravidin DyLight 650 (red), indicating co-localization of biotinylated proteins with AT8-positive structures. Co-localized pixels are highlighted in yellow in the merged image and in white in the “co-localization” images generated using the “Colocalization Threshold”) ImageJ plugin. **(B)** Western blots of the tissue lysates revealed higher level of fluorescent streptavidin signal in samples treated by AT8 ProPPr (AT8+) compared to negative control were staining with AT8 antibodies was omitted (AT8-). **(C)** Ratios between neutravidin intensity in AT8+ and AT8- samples for each of the studied cases measured by Western blotting. **(D)** Scatterplot illustrating relationship between AT8+/AT8- signal ratio and tau burden quantified by digital pathology. **(E)** Scatterplot illustrating relationship between AT8+/AT8- signal ratio and number of identified proteins in AT8+ samples. **(F)** Control frontal cortex immunostained with AT8 phospho-tau antibody and developed with DAB., along with fluorescent neutravidin staining after proximity biotinylation in control frontal cortex with AT8 ProPPr. Abbreviations: AD, Alzheimer’s disease; PiD, Pick’s disease; CBD, corticobasal degeneration; PSP, progressive supranuclear palsy; CTRL, control.


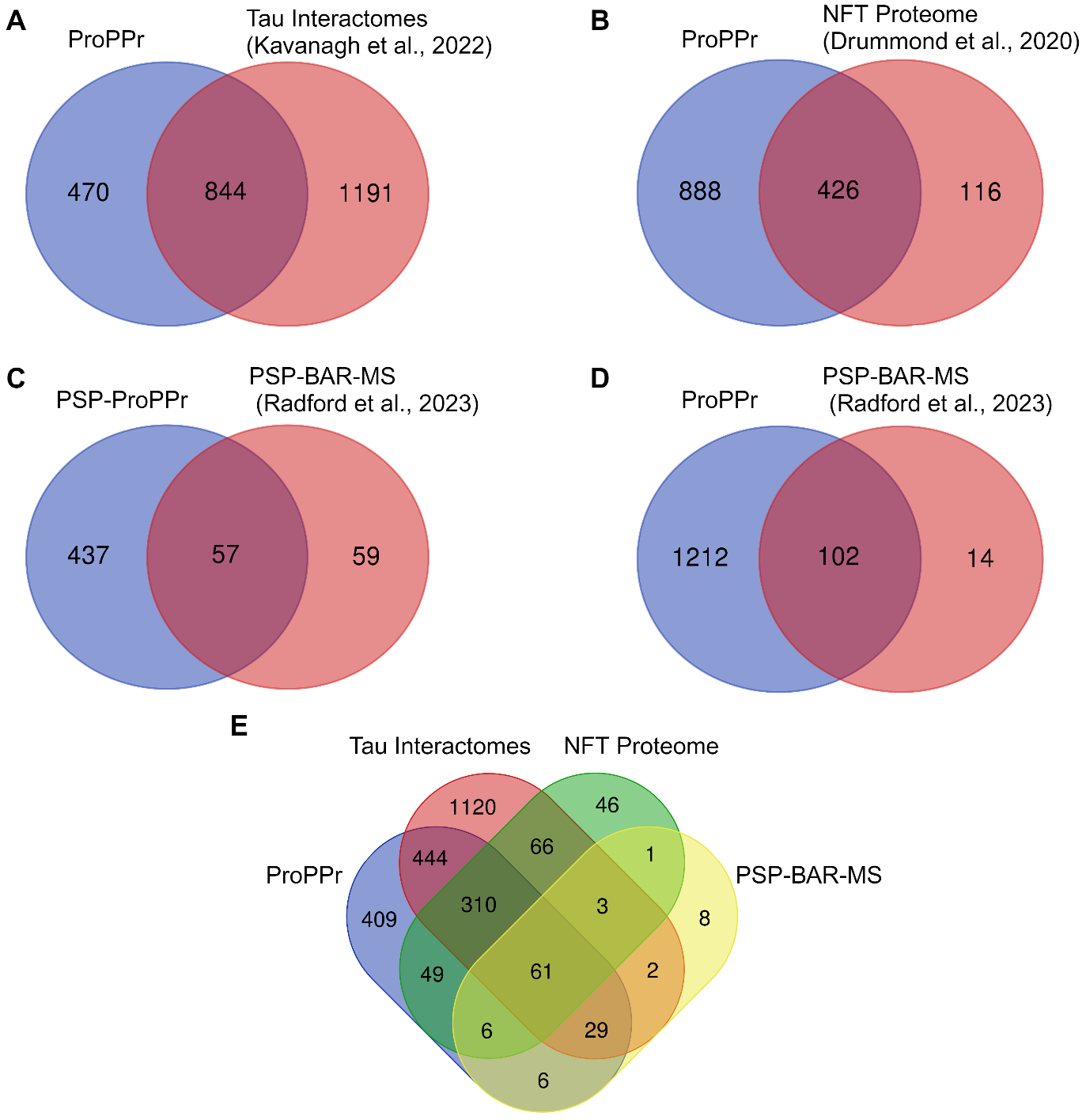


**Supplementary Fig. 3 ⎪ Comparison of the obtained set of phospho-tau associated proteins with previously published data**. **(A)** Venn Diagram for the overlap between our set of phospho-tau-associated proteins and tau interactors reported in previous studies (49). **(B)** Venn Diagram for the overlap between our set of phospho-tau-associated proteins and the proteome of NFTs in AD (50). **(C)** Venn Diagram for the overlap between our set of phospho-tau-associated proteins in PSP and the PSP-associated proteome examined by BAR-MS method (60). **(D)** Venn Diagram for the overlap between our set of phospho-tau-associated proteins found across the tauopathies and the PSP-associated proteome examined by BAR-MS method (60). **(E)** Venn Diagram showing overlap between our set of phospho-tau-associated proteins and the other published tau-associated protein sets. Abbreviations: ProPPr, probe dependent proximity profiling; BAR, biotinylation by antibody recognition; MS, mass-spectrometry; PSP, progressive supranuclear palsy; AD, Alzheimer’s disease; NFTs, neurofibrillary tangles.


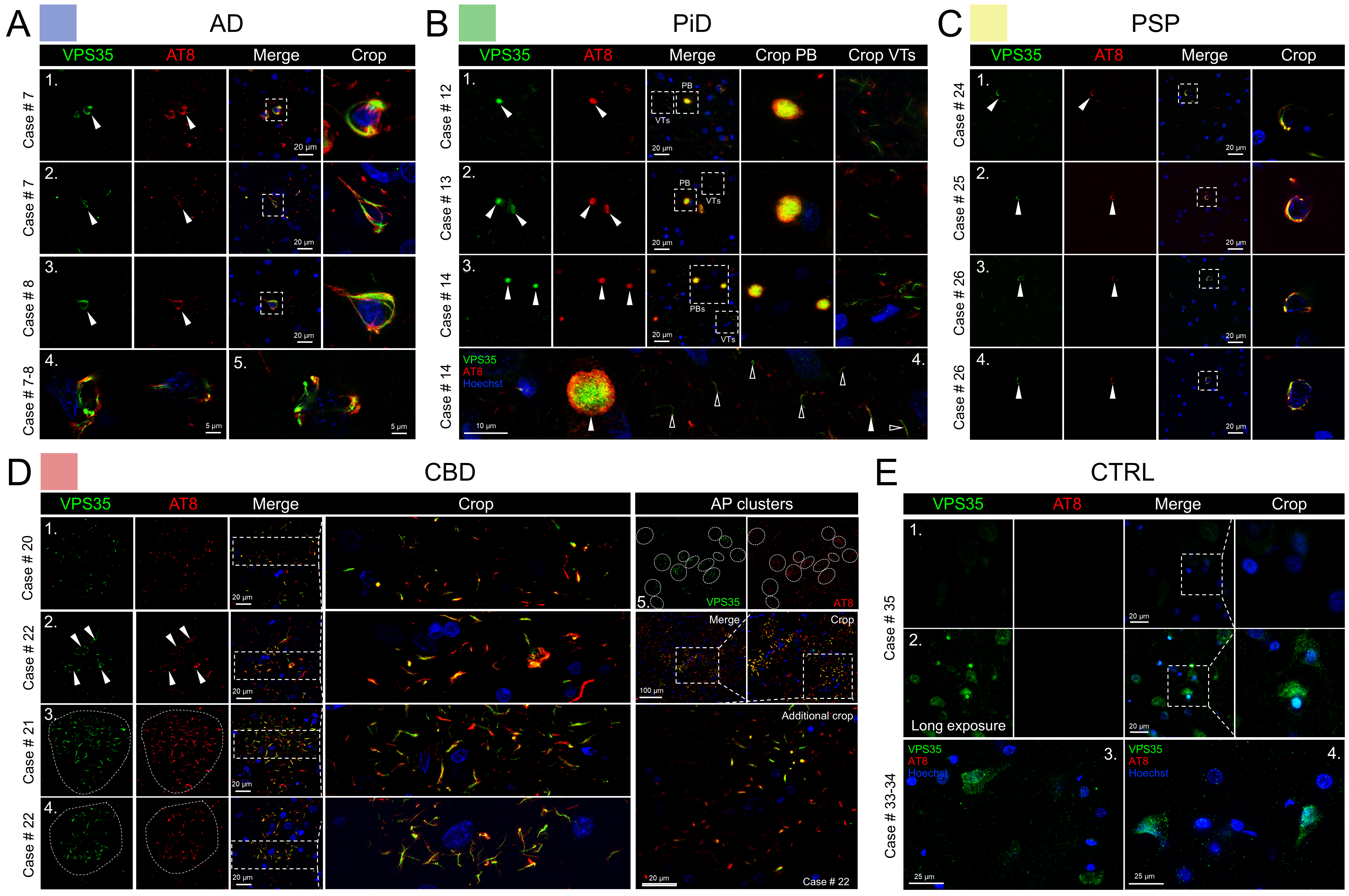


**Supplementary Fig. 4 | VPS35 distribution in frontal cortices from tauopathy and control cases. (A)** VPS35 was sequestered into neuronal tangle pathology (series 1-3) across all AD cases examined, including pre-tangle pathology (series 4-5). **(B)** VPS35 highlighted the cores of AT8 positive PBs of various sizes across all PiD cases, often in proximity to VPS35-positive thread structures (VTs), shown in cropped images (series 1-4). VPS35 threads were found to be predominantly AT8-negative or partially positive (series 5, unfilled arrowheads denote co-localization areas on VTs, white arrowheads show VPS35 only threads). **(C)** PSP CBs of various sizes present in each frontal cortex section from all cases analyzed co-localized with sequestered VPS35 protein (series 1-4). **(D)** APs of differing densities sequester VPS35 (series 1-4, established plaques are outlined with a dashed circle in series 3 and 4). The distribution of APs and engagement of VPS35 with phospho-tau in highly affected cortical areas displays the robustness of the pathologic relationship (series 5). **(E)** VPS35 and AT8 immunostaining in frontal cortex from neuropathologically unaffected control cases. To examine non-pathologic VPS35 distribution, the exposure for control cases required longer times, due to the higher intensity of abnormal VPS35 in disease tissue (series 1 – 60 ms exposure, series 2-4 – 200 ms exposure). Abbreviations: VPS35, vacuolar protein sorting-associated protein 35; AD, Alzheimer’s disease; PiD, Pick’s disease; CBD, corticobasal degeneration; PSP, progressive supranuclear palsy; CTRL, control; PTs, pre-tangles; NFTs, neurofibrillary tangles; APs, astrocytic plaques; CBs, coiled bodies; PBs, Pick bodies; VTs, VPS35 threads; NTs, neuropil threads.


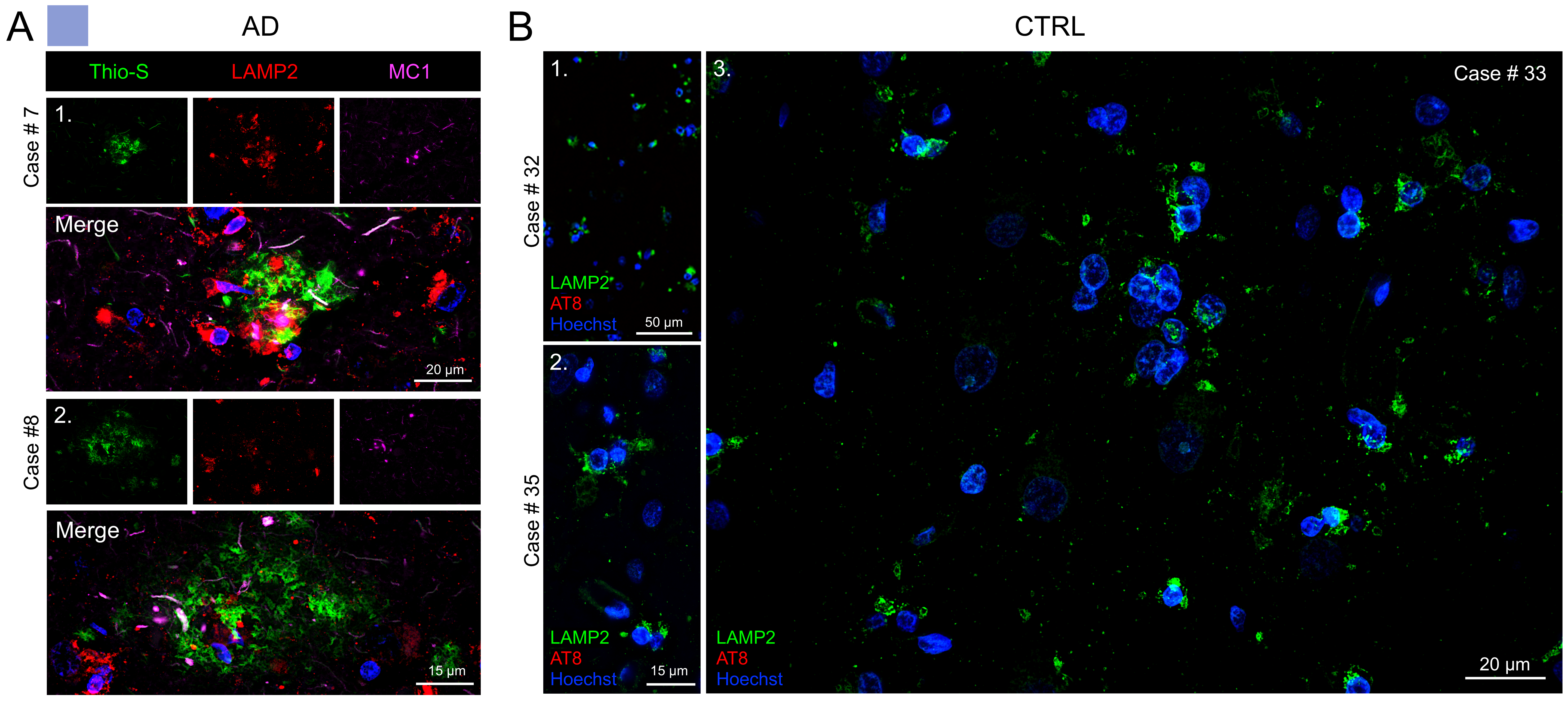


**Supplementary Fig. 5 | LAMP2 distribution in frontal cortices from AD and control cases. (A).** Co-staining of AD frontal cortices with thioflavin-S, MC1 anti-tau conformation-specific antibody and anti-LAMP2 antibody confirmed that LAMP2 abnormally accumulates in NPs (series 1-2). **(B)** Immunostaining of control cases with anti-LAMP2 antibody demonstrated cytosolic and vesicular LAMP2 localization within the soma and some cellular processes (series 1-3).


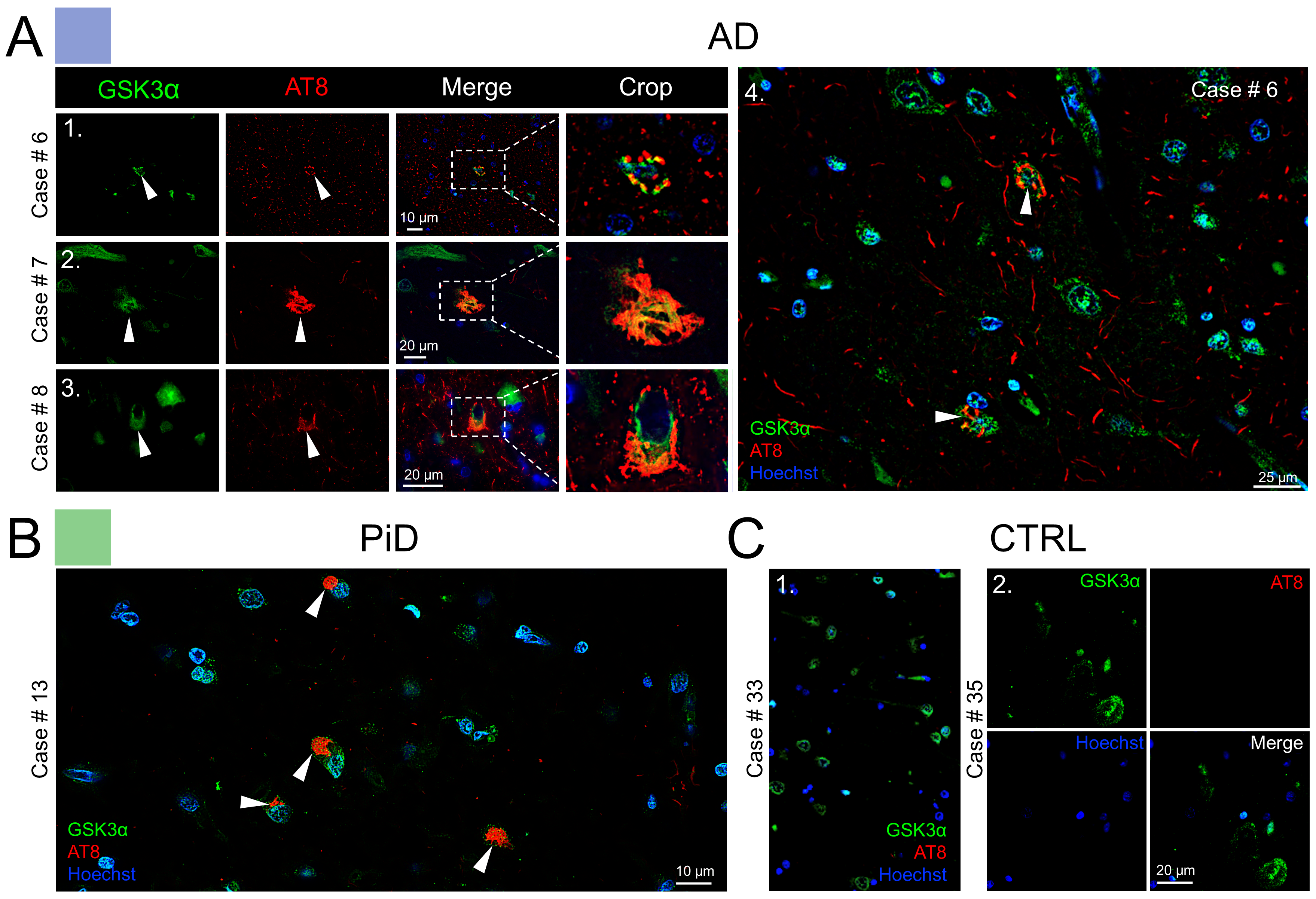


**Supplementary Fig. 6 | Distribution of GSK3α in AD, PiD and control cases. (A)** Neuronal pre-tangles and NFTs in AD show co-localized signal of AT8 and GSK3α across all cases (series 1-4). **(B)** Tile image showing the abundance of GSK3α-positive PBs in PiD. **(C)** GSK3α localization in unaffected control cases. Abbreviations: LAMP2, lysosome-associated membrane glycoprotein 2; AD, Alzheimer’s disease; CTRL, control; NPs, neuritic plaques.


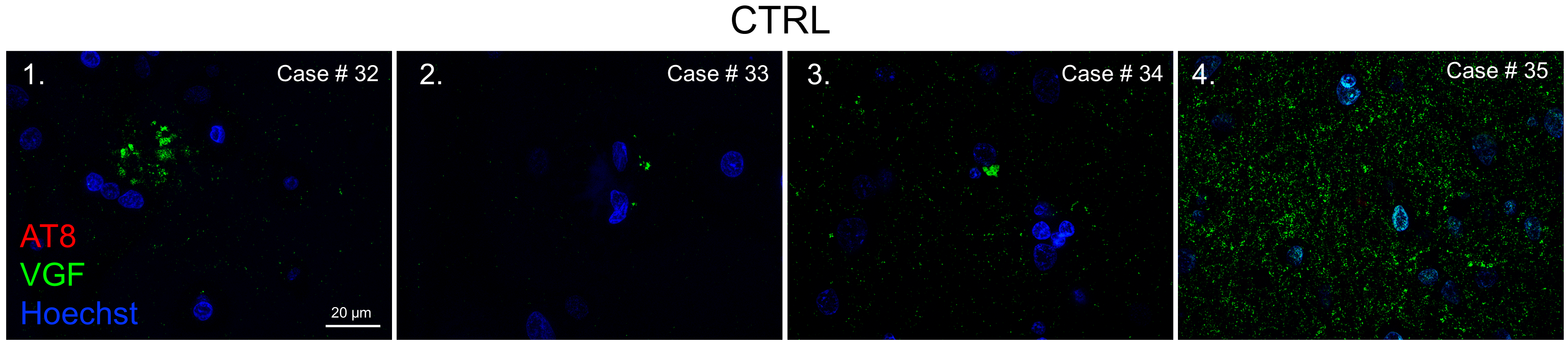


**Supplementary Fig. 7 | VGF localization in frontal cortices from control cases.** The distribution of VGF observed in control tissue, lacking tau pathology in the frontal cortex, from age-matched cases revealed that VGF punctate signal was sporadically clustered across the tissue (series 1-3). In an exceptionally young case (series 4) the VGF signal was markedly higher, with greater levels of diffuse punctate signal throughout the cortex, under the same imaging parameters. Abbreviations: CTRL, control.


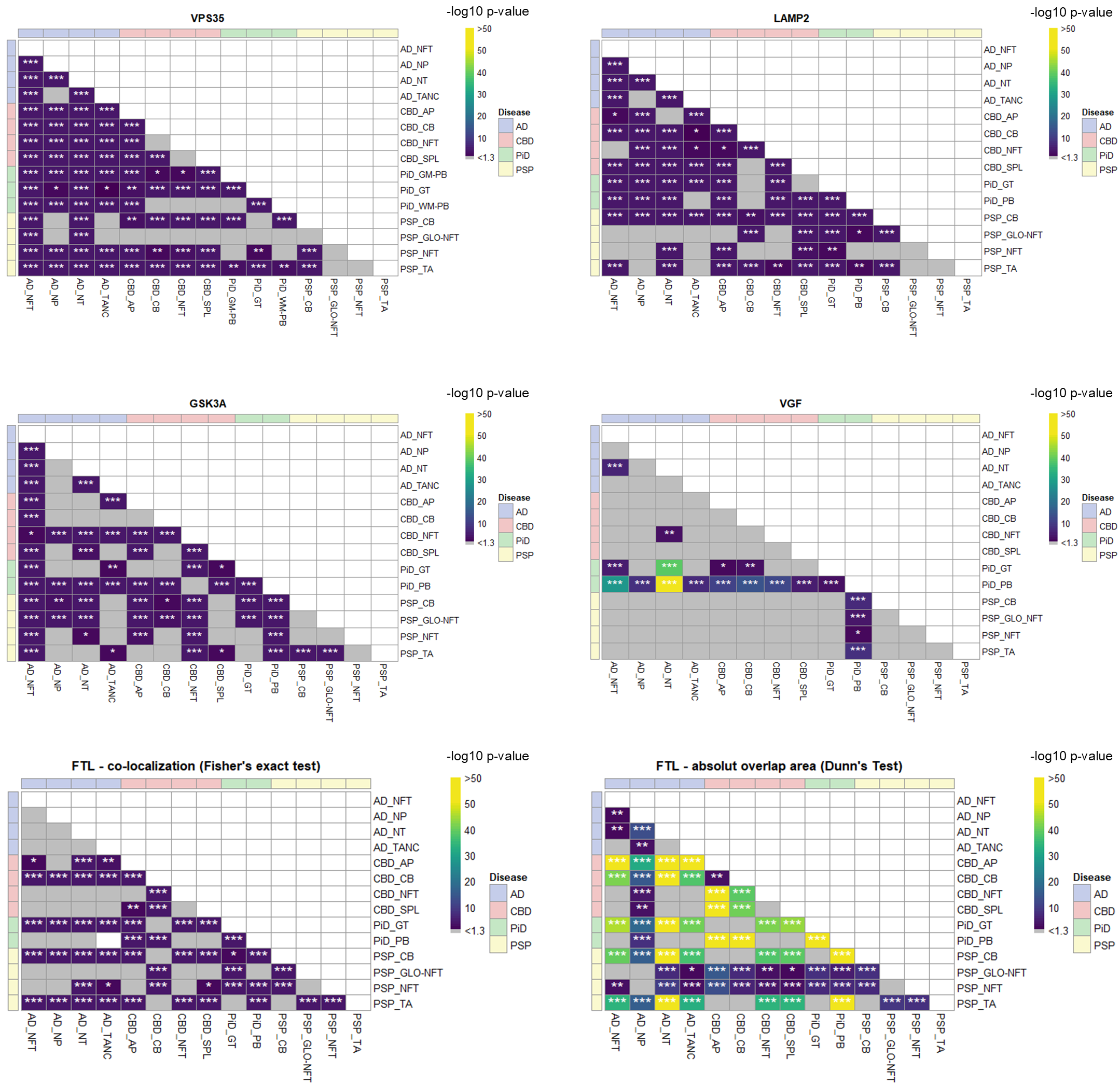


**Supplementary Fig. 8 | Statistical analysis of quantified co-localizations.** The results of the pairwise statistical comparisons of co-localization frequencies between major phospho-tau lesion categories are plotted as heatmaps. For VPS35 (data from the Fig. 4F, pie charts), LAMP2 (data from the Fig. 5E, pie charts), GSK3α (data from the Fig. 7D, pie charts), VGF (data from the Fig. 8E, pie charts) and FTL (data from the Fig. 9E, pie charts) the comparisons were performed using pairwise Fisher’s exact test followed by Bonferroni correction for multiple comparisons. In addition, for FTL (data from the Fig. 9F, violin plots) we compared absolute areas of overlap for individual lesions using Kruskal-Wallis test and post hoc Dunn’s test (lower right panel). Each cell represents a pairwise comparison (row vs column). Significant differences are color-coded from purple to yellow based negative logarithm of adjusted p-values, as indicated on the heatmap legends. Grey cells indicate absence of statistical significance. * - adj. p-value <0.05; ** - adj. p-value < 0.01, *** - adj. p-value < 0.001.
