# Supplementary material for: Probe-dependent Proximity Profiling (ProPPr) Uncovers Similarities and Differences in Phospho-Tau-Associated Proteomes Between Tauopathies
Source: Mol Neurodegener. 2025 Mar 13;20:32. doi: 10.1186/s13024-025-00817-0 (PMC11905455; doi:10.1186/s13024-025-00817-0)
Supplement: Supplementary file 2 — Supplementary Material 2. [file 13024_2025_817_MOESM2_ESM.docx]

**Supplementary Table 1.** Antibody combinations for human tissue validation using frontal cortex from AD, PiD, PSP, CBD and control cases.

| Primary target | Host species | Isotype | clonality | Secondary | AFP | Antibody form | Image  Color | Vendor |
| --- | --- | --- | --- | --- | --- | --- | --- | --- |
| AT8 phospho-tau and candidate double labelling | | | | | | | | |
| Phospho-tau (S202/S205), clone AT8, ThermoFisher MN1020 | Mouse | IgG | monoclonal | Goat anti-mouse | 647 | F(ab’)2 | Red | ThermoFisher |
| VPS35 c-term clone C3, GeneTex, GTX108058 | Rabbit | IgG | polyclonal | Donkey anti-rabbit | 488 | F(ab’)2 | Green | ThermoFisher |
| LAMP2, abcam clone EPR19512, ab199946 | Rabbit | IgG | monoclonal | Donkey anti-rabbit | 488 | F(ab’)2 | Green | ThermoFisher |
| VGF, abcam clone EPR2702973, ab308287 | Rabbit | IgG | monoclonal | Donkey anti-rabbit | 488 | F(ab’)2 | Green | ThermoFisher |
| GSK3a, abcam cline EP793Y, ab40870 | Rabbit | IgG | monoclonal | Donkey anti-rabbit | 488 | F(ab’)2 | Green | ThermoFisher |
| FTL (Novus, NBP2-34072) | Rabbit | IgG | polyclonal | Donkey anti-rabbit | 488 | F(ab’)2 | Green | ThermoFisher |
| LAMP2, MC1 and This staining in in AD cases | | | | | | | | |
| MC1 (Peter Davies, conformational tau) | mouse | IgG1 | monoclonal | Goat anti-mouse, H+L, highly cross absorbed | 647 | F(ab’)2 | Magenta | ThermoFisher, A48289 |
| LAMP2 abcam clone EPR19512, ab199946 | rabbit | IgG | monoclonal | Goat anti-rabbit, H+L, highly cross absorbed | 555 | F(ab’)2 | Red | ThermoFisher, A-21430 |
| Triple labelling with cell type specific markers in CBD (Fig. 10) | | | | | | | | |
| FTL (novus, NBP2-34072) | rabbit | IgG | polyclonal | Goat anti-rabbit, H+L, highly cross absorbed | 488 | F(ab’)2 | Green | ThermoFisher, A48282 |
| GFAP clone 2.2B10,13-0300 | rat | IgG2a, kappa | monoclonal | Goat anti-rat, H+L, preabsorbed | 555 | F(ab’)2 | Red | Abcam, ab150162 |
| IBA1, abcam, ab300156 | rat | IgG2a | monoclonal | Goat anti-rat, H+L, preabsorbed | 555 | F(ab’)2 | Red | Abcam, ab150162 |
| Phospho-tau (S202/S205), clone AT8, ThermoFisher MN1020 | mouse | IgG1, kappa | monoclonal | Goat anti-mouse, H+L, highly cross absorbed | 647 | F(ab’)2 | Magenta | ThermoFisher, A48289 |

Abbreviations: AFP, AlexaFluor Plus.
